# Compact Disc-Derived Nanocarbon-Supported Catalysts with Extreme Catalytic Activity

**DOI:** 10.1021/acsami.4c17754

**Published:** 2025-01-22

**Authors:** Chia-Hung Lin, Yi-Jui Yeh, Tzu-Hsiang Chien, Shao-Yu Chen, Loganathan Veeramuthu, Chi-Ching Kuo, Kuo-Lun Tung, Wei-Hung Chiang

**Affiliations:** † Department of Chemical Engineering, National Taiwan University of Science and Technology, Taipei 10607, Taiwan; ‡ Department of Chemical Engineering, National Taiwan University, Taipei 10607, Taiwan; § Institute of Organic and Polymeric Materials, Research and Development Center of Smart Textile Technology, National Taipei University of Technology, Taipei 10608, Taiwan; ∥ Advanced Research Center for Green Materials Science and Technology, National Taiwan University, Taipei 10607, Taiwan; ⊥ Sustainable Electrochemical Energy Development (SEED) Center, National Taiwan University of Science and Technology, Taipei City 10607, Taiwan

**Keywords:** Plasma, Synthesis, Catalysts, Plastics recycling, Pollutant reduction, Nanotechnology

## Abstract

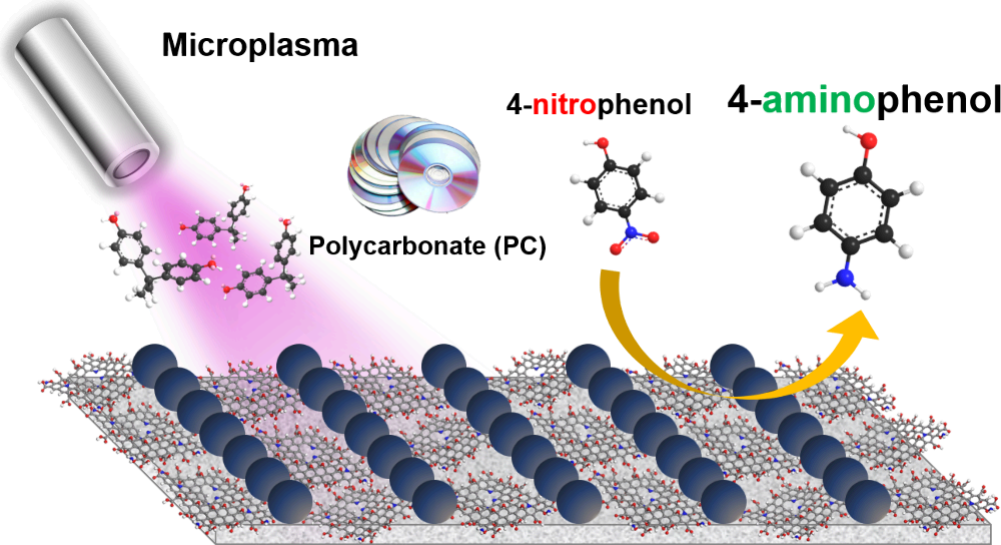

Advanced carbon–metal hybrid materials with controllable electronic and optical properties, as well as chemical reactivities, have attracted significant attention for emerging applications, including energy conversion and storage, catalysis and environmental protection. However, the commercialization of these materials is hampered by several vital problems, including energy-intensive synthesis and expensive chemicals, and inefficient control of their structures and properties. Herein, we report the simple and controllable engineering of nanocarbon–metal self-assembled silver nanocatalysts (SSNs) derived from polycarbonate (PC)-based optical discs using microplasmas under ambient conditions. The plasma-engineered catalysts exhibited controlled properties including surface functionalities, hydrophilicities, Ag^+^/Ag^0^ metallic states, and Ag loading. The synthesized catalysts leverage localized surface plasmon resonance (LSPR) properties, enabling enhanced catalytic activity for the rapid reduction of carcinogenic 4-nitrophenol (4-NP) to the valuable pharmaceutical intermediate 4-aminophenol (4-AP), achieving a high reaction rate constant of 0.2 ± 0.0 s^–1^ and completing the reduction in just 30 s. Demonstrating robust performance, the SSNs maintained up to 90% conversion efficiency after ten recycling cycles, underscoring their stability and reusability. This work not only presents an effective approach to upcycling optical disc waste, but also highlights the potential of plasma-engineered nanocatalysts in environmental remediation, offering a low-energy solution for high-efficiency pollutant reduction.

## 1 Introduction

Nanocarbon-supported silver nanoparticles (Ag NPs) have attracted significant attention because of their unique physicochemical properties, including exceptional antimicrobial activity, electrical conductivity, and pronounced localized surface plasmon resonance (LSPR) effects.^1,2^ The LSPR phenomenon, which arises from the collective oscillation of surface electrons in response to incident light, imparts Ag NPs with enhanced light absorption, scattering, and catalytic capabilities, rendering them highly effective in applications spanning biosensing, imaging, and environmental catalysis.^3−6^ Ag NPs have demonstrated efficacy in catalyzing the reduction of organic pollutants, such as the conversion of 4-nitrophenol (4-NP) to 4-aminophenol (4-AP), a valuable pharmaceutical intermediate, under mild reaction conditions.^7−9^ A key advantage of these nanostructures is the synergistic supported effect provided by the carbon-based materials.^10−12^ Carbon supports facilitate charge transfer between AgNPs and the support, improving analyte adsorption, conductivity, and overall catalytic performance.^13,14^ Moreover, these carbon supports provide a stable surface for nanoparticle adhesion, which is important for catalyst recyclability because it helps prevent nanoparticle aggregation and loss during repeated use.^15,16^ However, the preparation of porous Ag nanostructures on substrates faces several key challenges, including time-consuming processes, multiple preparation steps, use of toxic chemicals, and costly Ag precursors. These factors limit the efficiency and sustainability of the current methods.^17,18^

Polycarbonate (PC), a highly versatile thermoplastic polymer, is extensively employed in diverse applications, including optical discs (ODs), food packaging, safety equipment, automotive components, and construction materials, because of its outstanding mechanical strength, thermal stability, and optical clarity.^19−22^ Existing recycling methodologies for PC waste, including mechanical and chemical processes, are fraught with significant drawbacks that impede their widespread adoption.^23,24^ Mechanical recycling involves physical processing of waste discs through separation, cleaning, and shredding, resulting in the recovery of reusable plastic particles. However, this approach is hampered by the degradation of polymer properties and the inadvertent generation of microplastics, which are defined as particles smaller than five millimeters and are notorious for their persistence in the environment and potential adverse effects on human health.^25−27^ Chemical recycling, on the other hand, involves the depolymerization of PC waste via solvent dissolution and high-temperature treatment, with the aim of recovering monomers or other valuable chemical intermediates. However, this process often releases hazardous compounds, such as bisphenol A (BPA), a known endocrine disruptor and carcinogen,^28^ compounding the environmental and health concerns associated with PC waste. Furthermore, both recycling routes are further complicated by the need to remove the metal memory layer, which is typically composed of silver embedded within optical discs. This additional step involves complex purification processes that are energy-intensive, costly, and environmentally detrimental. These limitations underscore the urgent need for innovative recycling strategies that are not only efficient and cost-effective, but also capable of minimizing environmental impacts by operating under ambient conditions without generating harmful byproducts.

**Scheme 1:**
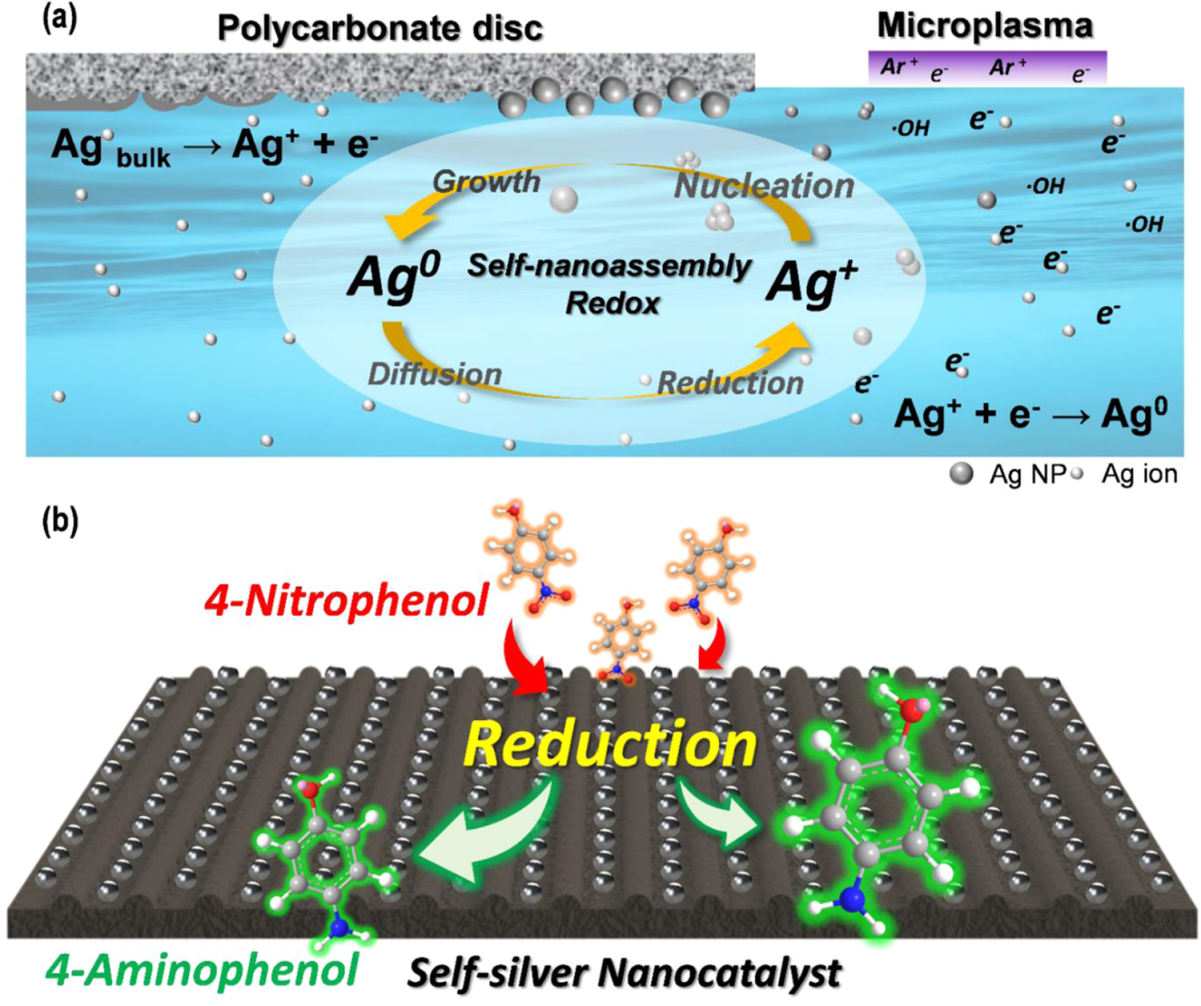
Microplasma Synthesis Nanocarbon-Supported Self-Assembled Silver Nanocatalysts (SSNs) with Extreme Hydrogenation Activity (a) Schematic representation of the synthesis of SSNs from PC optical discs using microplasmas at ambient conditions. The process involves the *in situ* reduction of Ag^+^ ions to Ag^0^ nanoparticles through a self-assembly redox mechanism facilitated by microplasma-generated reactive species under ambient conditions. This approach promotes the nucleation, growth, diffusion, and reduction of Ag, resulting in highly active plasmonic Ag^0^ nanoparticles embedded within a carbonaceous matrix. (b) Catalytic reduction pathway of 4-NP to 4-AP using synthesized SSNs. The SSNs efficiently facilitated the reduction reaction owing to their optimized surface properties, high electron transfer capability, and enhanced catalytic sites, significantly improving the pollutant reduction efficiency.

In this work, we present a novel one-step upcycling approach utilizing atmospheric-pressure microplasma technology to convert waste PC optical discs into nanocarbon-supported self-assembled plasmonic silver nanocatalysts (SSNs) under ambient conditions. Microplasma engineering is a useful and effective technique to synthesis nanomaterials including metal nanoparticles, semiconductor quantum dots and nanocomposites.^29−33^ Other nanoparticle synthesis methods often involve high temperatures, complex multistep purification, and solvent-intensive processes. Conventional methods such as chemical reduction,^34^ thermal decomposition,^35^ and hydrothermal synthesis^36^ typically demand substantial energy input and produce significant chemical waste, raising environmental and economic concerns. In contrast, this plasma-assisted process circumvents the high-temperature, multistep purification, and solvent-intensive requirements of conventional recycling methods, offering a sustainable and scalable alternative that significantly reduces environmental impact. The microplasma treatment facilitated the *in situ* reduction of silver ions (Ag^+^) and their subsequent self-assembly into metallic Ag^0^ nanoparticles within a carbonaceous material derived from the PC substrate (Scheme 1a and Figure S1). The plasma-engineered SSNs exhibit distinctive and tunable surface functionalities, characterized by a high hydroxyl group content (46.35–58.11%) and a stable negative zeta potential of −60 mV, significantly enhancing their hydrophilicity and electron transfer efficiency. The precisely controlled Ag^+^/Ag^0^ ratio (3.996–2.818) and Ag loading (0.0875–0.04) across the SSN_5_–SSN_20_ series optimize the balance between catalytic activity and structural stability, enabling the rapid catalytic reduction of 4-NP to 4-AP with a high reaction rate constant of 0.2 ± 0.0 s^–1^, completing the reaction in just 30 s. Notably, the SSNs maintained their catalytic performance over multiple cycles, retaining up to 94% of their initial conversion efficiency after ten reuses, underscoring their stability and potential for long-term catalytic applications (Scheme 1b). This study not only demonstrates a transformative approach to PC waste management, but also shows the potential of plasma-engineered nanocatalysts as multifunctional materials for environmental remediation. By converting waste into high-value catalytic materials, this study paves the way for a new paradigm in sustainable recycling and upcycling, offering an integrated solution to the dual challenges of plastic pollution and the need for advanced functional materials in environmental and chemical engineering applications.

## 2 Experimental Section

### 2.1 Synthesis of Nanocatalysts

SSNs were synthesized in an electrochemical reactor under ambient conditions using direct current (DC) argon (Ar) microplasmas. The reactor passed 25 sccm (standard cubic centimeter per minute) of Ar gas through a stainless-steel hollow capillary tube as the cathode (inner diameter: 178 μm). The anode prepared the disc (2.8 × 2 cm^2^) from the waste polycarbonate disc. Prior to use, the disc was soaked in deionized water (DIW) and cleaned for 5 min using the ultrasonic cleaner. The distance between the cathode and anode disc was precisely calibrated to 2 cm. To start the reaction, 1 M sodium hydroxide was added to the PTFE reactor as the electrolyte, allowing the plasma and anode disc (2 × 2 cm^2^) to be in contact with the liquid surface. The plasma was maintained using a DC power source (UDC 5N30 (150 W), Hung Hui Technology, Taiwan) connected to a 250 kΩ ballast resistor. The reaction was controlled under the current limiting condition of 10 mA for 5–20 min to synthesize SSN under ambient conditions. After the plasma process, the SSN composite was rinsed with deionized water and dried under ambient conditions.

### 2.2 Catalytic Study of Nanocatalysts

Catalytic reduction tests were conducted for the reduction of 4-NP and dye degradation. For each test, 3 mL of a 1 mM 4-NP or dye solution was mixed with 3 mL of an excess reducing agent, NaBH_4_ (0.1 M), in a sample vessel. In the 4-NP reduction experiment, the color of the solution immediately changed from pale to deep yellow. The synthesized SSN samples (2 × 2 cm^2^) were cut into 16 fragments (0.5 × 0.5 cm^2^) for the experiments. Each SSN fragment (0.5 × 0.5 cm^2^) was immersed in the respective mixed solution. To analyze the reduction performance, 0.5 mL of the solution was taken every 30 s, mixed with deionized water (1.5 mL), and measured using UV–vis absorption spectroscopy.

### 2.3 Reusability and Stability Study of Nanocatalysts

After the reaction, the as-synthesized samples were rinsed with DI water several times and dried under typical conditions for 30 min before the recycling tests were repeated. The subsequent reactions followed a similar procedure as in the previous tests.

## 3 Results and Discussion

### 3.1 Synthesis and Characterization of Nanocatalysts

**Figure 1:**
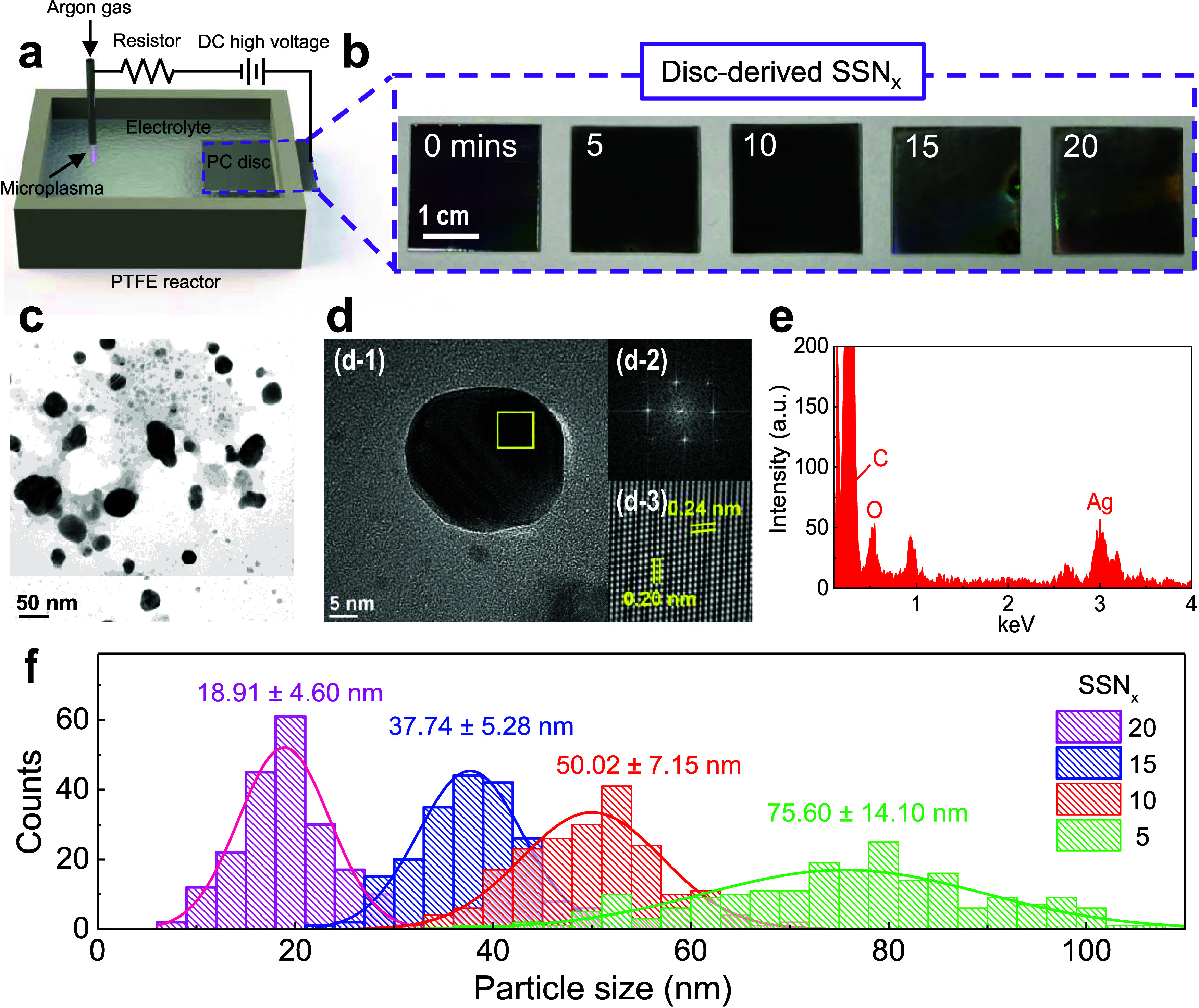
Synthesis and TEM analysis of plasma-engineered nanocatalysts. (a) Schematic of the microplasma reactor. (b) Photographs of samples treated with different plasma reaction times. (c) TEM image, (d-1) HRTEM image, (d-2) corresponding diffraction pattern, (d-3) inverse FFT image, and (e) EDX spectrum of synthesized SSN_10_ catalysts. (f) Size distributions of the SSN catalysts synthesized with different plasma treatment times.

**Figure 2:**
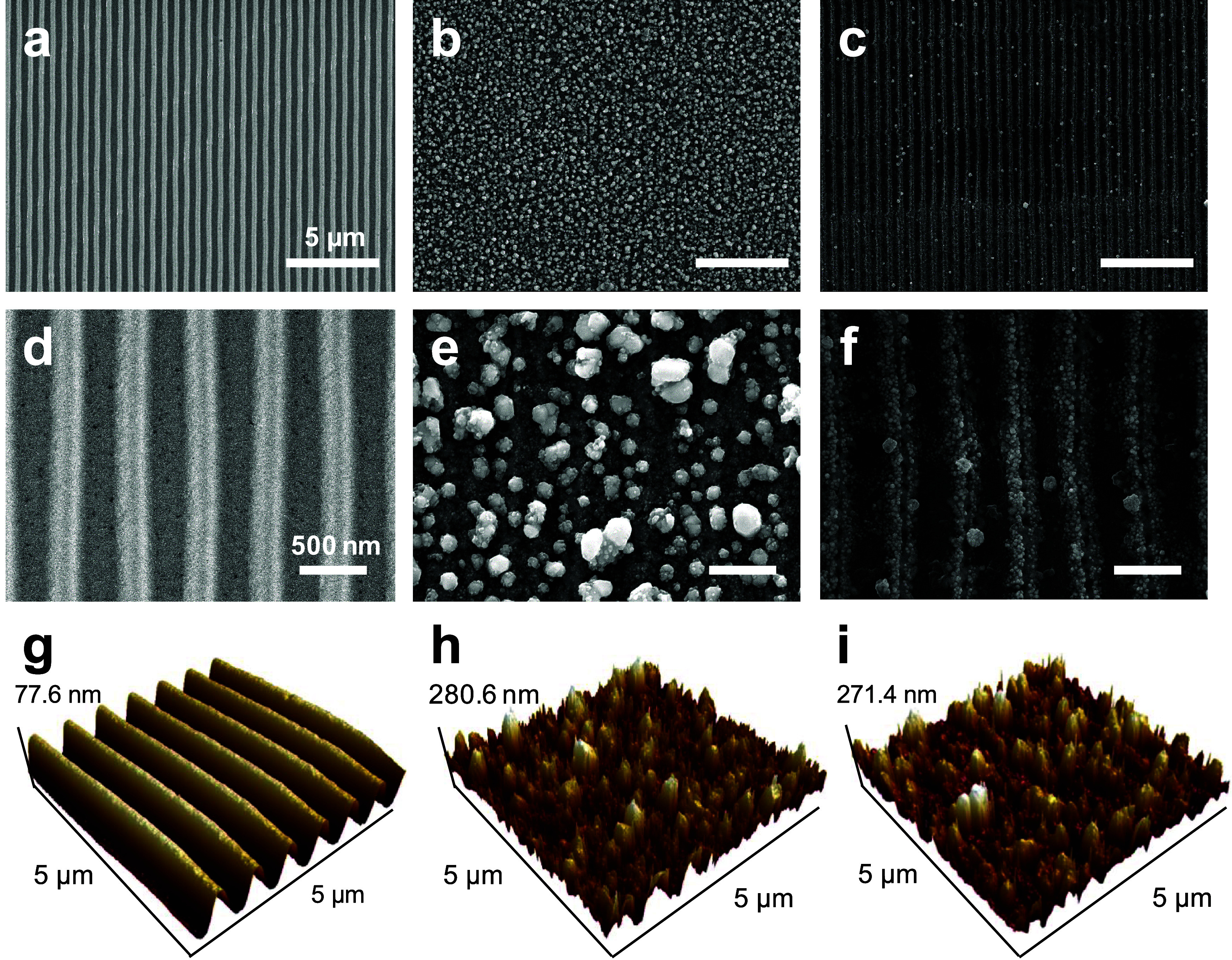
SEM and AFM analyses of SSNs. SEM images at low magnification of (a) the waste disc, (b) SSN_10_, and (c) SSN_20_. (Scale bar: 5 μm). High-magnification SEM images of (d) waste disc, (e) SSN_10_, and (f) SSN_20_. (Scale bar: 500 nm). AFM images of (g) the waste disc, (h) SSN_10_, and (i) SSN_20_.

**Figure 3:**
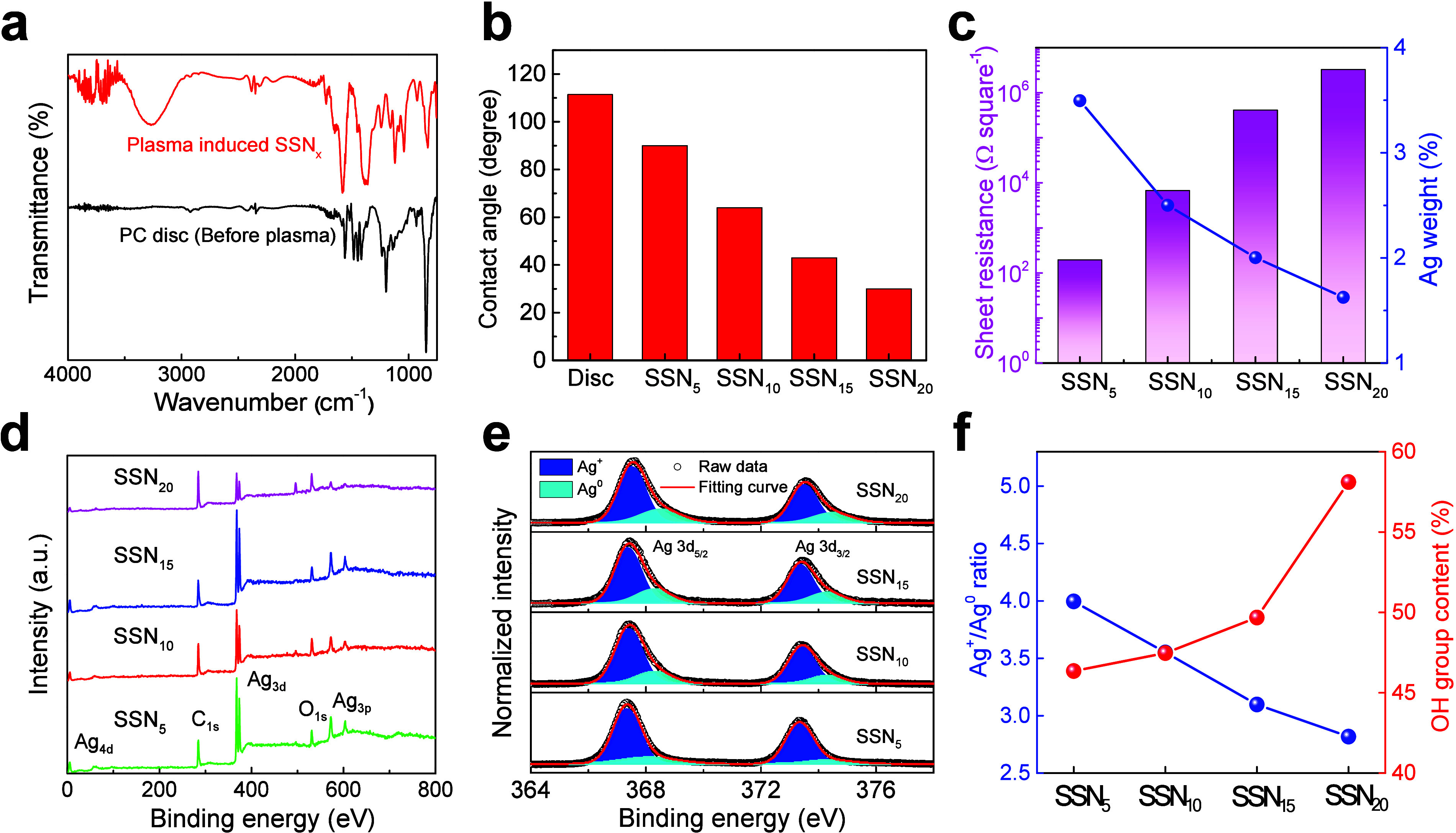
Comprehensive analysis studies of nanocatalysts. (a) FTIR spectra, (b) contact angle, (c) electrical resistance and catalyst quantity, and (d) XPS survey spectra of different SSNs prepared with different plasma reaction times. (e) HRXPS spectra of Ag 3d region. (f) Ag state ratio and OH group analysis.

Figure 1(a) presents a schematic of the experimental setup, including the plasma generation region and waste disc as the cathode. This setup is critical for the formation and deposition of nanoparticles, demonstrating the capabilities of the microplasma technology in synthesizing nanomaterials under ambient conditions. Figure 1(b) shows a series of photographs of the SSNs derived from disc materials subjected to varying plasma reaction times (0, 5, 10, 15, and 20 min). The images visually depict the impact of plasma exposure on the physical and surface properties of the SSNs, with clear changes in color and texture observed as the reaction time increased. These observations suggest significant alterations in the nanoparticle morphology and composition, which are directly influenced by the duration of plasma treatment. The structural morphology of the SSNs is further explored in Figure 1(c) and S2 using transmission electron microscopy (TEM). The TEM image reveals a polydisperse system with nanoparticles of various sizes, reflecting the degree of aggregation and dispersion within the nanocatalysts. A more detailed examination of the nanoparticle structure is provided in Figure 1(d) and S4, where high-resolution TEM (HRTEM) images offer a closer look at the individual SSN particles. Figure 1**(d-1)** shows distinct lattice fringes within the nanoparticles with polycrystalline phases, while Figure 1**(d-2)** shows the corresponding Fast Fourier Transform (FFT) pattern, confirming the crystalline nature of the silver nanoparticles. Figure 1**(d-3)**, an inverse FFT image, shows distinct lattice fringes with spacings of 0.24 ± 0.01 and 0.20 ± 0.01 nm, corresponding to the (111) and (200) planes of face-centered cubic (fcc) silver. These findings validate the crystallinity and specific lattice orientations of the synthesized Ag nanoparticles.^37^ Furthermore, Supporting Information S3, **Figure S3** and **S23** describes the nanocarbon supports of the samples.

Energy-dispersive X-ray (EDX) analysis was used to determine the elemental composition of the SSN. The elemental composition of the SSNs was analyzed using EDX, as shown in Figure 1(e). The EDX spectrum revealed distinct peaks corresponding to carbon (C), oxygen (O), and silver (Ag), confirming the successful incorporation of Ag into the material.^38^ These data support the effective integration of metallic nanoparticles into SSNs, enhancing their potential catalytic performance. Figure 1(f) illustrates the particle size distributions of the SSNs synthesized at different plasma reaction times. The histograms demonstrate a clear trend where increasing reaction time leads to larger and more uniform nanoparticles, with mean particle sizes growing from 18.91 ± 4.60 nm to 75.60 ± 14.10 nm. As mentioned above, the microplasma synthesis of SSNs is a highly versatile and efficient strategy for the precise control of nanoparticle characteristics, significantly enhancing their catalytic and environmental functionalities. The above analyses verified the successful fabrication of crystalline Ag NPs supported on nanocarbons, highlighting the potential of microplasma technology for carbon–metal hybrid materials preparation.

Figure 2 and S7–10 illustrates the morphologies of SSNs synthesized from waste PC optical discs using microplasma technology. Scanning electron microscope (SEM) images captured at various magnifications provide critical insights into the morphological evolution of the nanocatalysts during the synthesis process. In Figure 2(a) and (d) show the surface morphology of the original PC optical disc, displaying an orderly striped structure. These stripes were due to the inherent grooves of the disc, which exhibited a highly organized and evenly spaced pattern. These images (Figure 2**(b-c), (e-f)**) depict the waste PC discs after the plasma treatment. As shown in Figure 2(b) and (e), after 10 min of plasma reaction, numerous small silver particles begin to appear on the substrate surface and are uniformly distributed, forming an initial layer of silver nanoparticles. Further growth of these particles results in significantly larger and more uniformly spherical structures, enhancing the surface area and thereby improving the catalytic performance. Figures 2(c) and (f) present the disc after 20 min of treatment. The particles are more closely connected, forming a continuous film of nanoparticles with decreasing particle size and spacing between particles.

Figure 2**(g-i)** and S5 illustrate the surface morphology changes observed via atomic force microscopy (AFM) after different reaction times. Figure 2(g) depicts the sample before plasma treatment, whereas Figure 2(h) and **(i)** represent the sample morphology after 10 and 20 min of plasma treatment, respectively. Figure 2(g) shows the untreated sample. The surface had a well-defined, periodic structure with a reasonably smooth and uniform height of 77.6 nm. This baseline morphology served as a control, illustrating the initial state of the surface before any plasma-induced modification. After 10 min of plasma treatment (Figure 2(h)), the surface underwent significant roughening, reaching a height of 280.6 nm. Figure S6 shows this increase in surface roughness, as both roughness parameters (roughness average (Ra) and root-mean-square roughness (Rq)) reached their peak at this time. The substantial increase in surface roughness and height indicates that the plasma treatment effectively modifies the surface, creating a more complex and roughened morphology that maximizes the surface area, as confirmed by the peak surface area of 52.1 μm^2^ observed at this time point (Table S1). Continuing the plasma treatment for 20 min (Figure 2**(i)**) led to a small reduction in surface roughness, with a height of 271.4 nm. Surface area decreased from 52.1 μm^2^ at 10 min to 41.1 μm^2^ at 20 min, indicating stabilization. This could be due to structural coalescence or reactive site saturation. This process demonstrates the potential of this technology for fabricating efficient nanocatalysts from the initial orderly striped structure to the final continuous nanoparticle film. The structural changes not only enhance the catalytic performance of the material but also provide possibilities for other applications.

UV–vis absorbance spectroscopy and X-ray photoelectron spectroscopy (XPS) were used to investigate the synthesis and optical properties of the as-synthesized SSNs. Figure S11 show the absorbance spectra of as-synthesized SSNs generated using different plasma reaction times. Furthermore, COMSOL simulations were performed to determine the electric field distribution, providing Supporting Information to understand the material properties. Details of the simulation are provided in Supporting Information S4 and **Figure S12–15**.

The FTIR spectra shown in Figure 3(a), **Table S2** and Supporting Information S6. provide information on the functional groups found in the SSN. The significant peaks in the spectra are related to the different vibrational modes of the functional groups. The broad peak at approximately 3400 cm^–1^ indicates the presence of O–H stretching vibrations, which may be attributed to the adsorbed water or hydroxyl groups on the catalyst surface.^39^Figure 3(b) and Figure S19 show the contact angle measurements of the as-synthesized SSNs. Figure 3(c) and S16 shows the relationship between electrical resistance and catalyst quantity. The quantity of catalyst was determined by thermogravimetric analysis (TGA). This figure illustrates that, as the amount of catalyst increased, the electrical resistance decreased, indicating that a higher catalyst loading led to better conductivity. This tendency was predicted when the presence of more conductive Ag NPs decreased the total resistance of the catalyst material. This characteristic is essential for applications that require efficient electron transfer processes.

XPS survey spectra in Figure 3(d) and Table S3 show the elemental composition of the SSNs composite during different reaction periods. Peaks corresponding to Ag, C, and O were evident, confirming the incorporation of these elements into the catalyst structure. The intensity variations of these peaks with reaction time provide insights into the surface composition and possible changes due to plasma treatment. Figure 3(e) shows high-resolution XPS (HRXPS) spectra of the Ag 3d region for different reaction times. The spectra showed distinct peaks corresponding to Ag 3d_5/2_ and Ag 3d_3/2_. The binding energy positions and intensity ratios provided information on the oxidation state of silver in the catalyst. These details indicate modifications in the electronic structure and surface chemistry of Ag NPs, which are crucial for understanding their catalytic behavior. Figure 3(f) shows a detailed analysis of the Ag states and OH group content obtained from the HRXPS spectrum. The graph shows a quantitative analysis in which the Ag and OH groups are plotted against the reaction time. The findings show that increasing the reaction time increases both the Ag^0^ state and the OH group content. This indicated that the plasma process was a reduction reaction. Ag^+^ ions are converted to metallic Ag, while the number of surface-bound hydroxyl groups increases. Moreover, Figure S17, Table 1 and Supporting Information S7 details the surface chemical species and the contact angle measurements of the samples. The table presents the binding energy (B.E.), species, and contents of Ag 3d_5/2_ and O 1s.^40−42^ Notably, the silver statement ratio and contact angles varied with reaction time, providing further insight into the surface characteristics and hydrophilicity of the samples. The comprehensive characterization of SSN using various analytical techniques reveals significant insights into their structural, compositional, and functional properties. These discoveries have contributed to the optimization and application of the synthesized cost-effective (Section S8 and Table S5) SSN in various catalytic processes.

**Table 1:** Surface Chemical Composition and Wettability Analysis of SSNs Synthesized at Different Plasma Reaction Times

	Surface chemical species (Ag 3d_5/2_)				Surface chemical species (O 1s)			
Sample	B.E. (eV)	Species	Content (%)	Ag^+^/Ag^0^ ratio	B.E. (eV)	Species	Content (%)	Contact angle (degree)
SSN_5_	367.3	Ag^+^	47.30	3.996	529.2	Ag_2_O	6.288	90.5
	368.3	Ag^0^	11.84		530.9	C–OH	46.35	
					532.1	O=C—OH	34.58	
					533.2	C–O–C	13.08	
SSN_10_	367.3	Ag^+^	47.54	3.551	529.2	Ag_2_O	7.386	64.6
	368.3	Ag^0^	13.39		530.9	C–OH	47.47	
					532.1	O=C—OH	37.83	
					533.2	C–O–C	7.311	
SSN_15_	367.3	Ag^+^	44.61	3.098	529.2	Ag_2_O	4.753	43.7
	368.3	Ag^0^	14.40		530.9	C–OH	49.68	
					532.1	O=C—OH	35.07	
					533.2	C–O–C	10.50	
SSN_20_	367.3	Ag^+^	44.24	2.818	529.2	Ag_2_O	4.494	22.8
	368.3	Ag^0^	15.70		530.9	C–OH	58.11	
					532.1	O=C—OH	21.34	
					533.2	C–O–C	4.155	

### 3.2 Catalytic Study of Nanocatalysts

**Figure 4:**
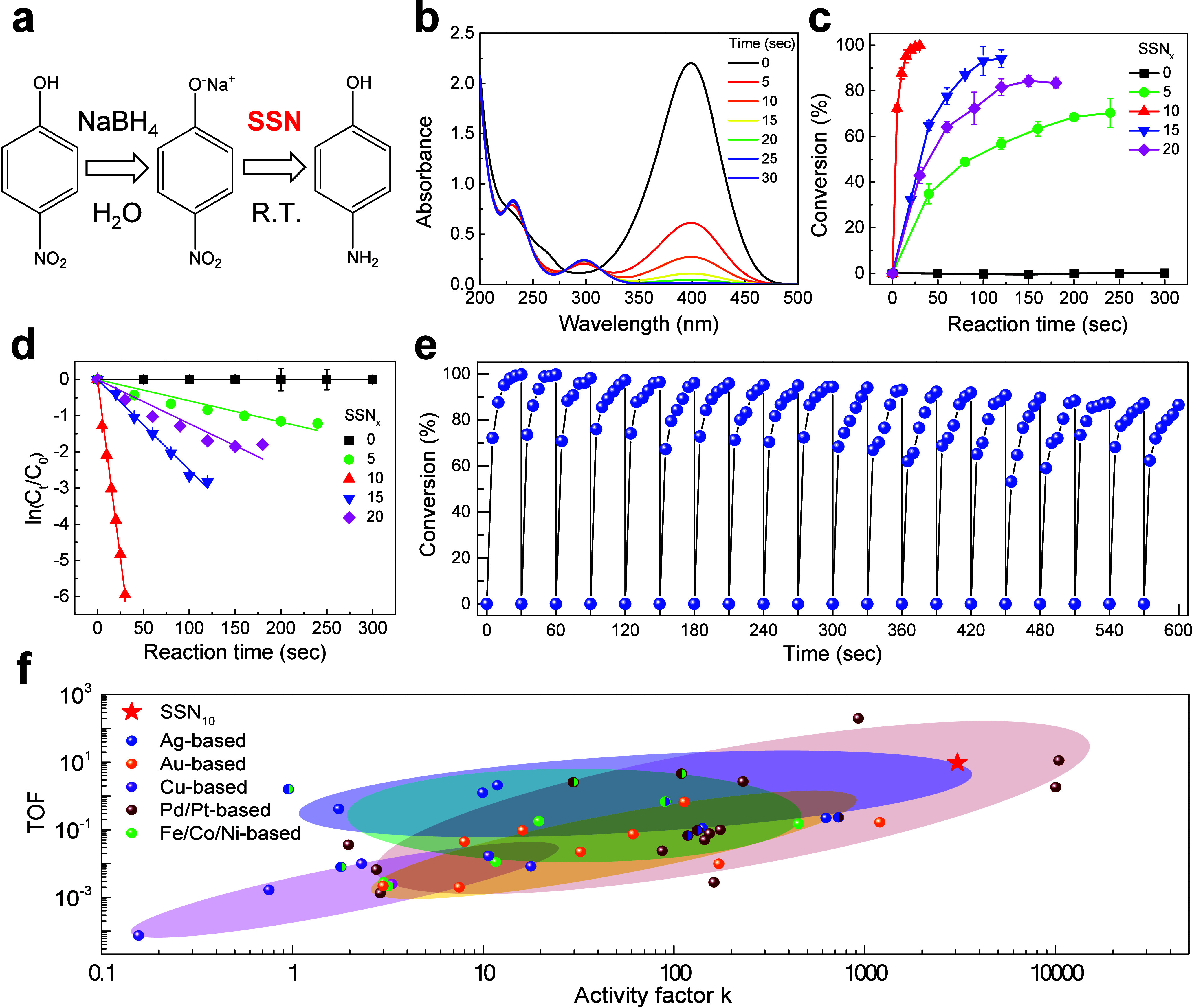
Catalytic studies of nanocatalysts. (a) Reaction pathway of 4-NP reduction. (b) UV–vis absorption spectra of the reduction of 4-NP. (c) Conversion rates and (d) reaction kinetics with first-order reactions of different SSN_*x*_, where *x* = 0, 5, 10, 15, and 20. (e) Catalyst reusability for 4-NP reduction. (f) Comparison of the catalytic performance of SSN_10_ and previously reported catalysts for 4-NP reduction.

Hydrogenation plays a crucial role in numerous fields, particularly in environmental applications. Hydrogenation aids in the conversion of hazardous molecules into less toxic forms, making it useful for procedures such as organic pollution removal. In this study, we evaluated the catalytic hydrogenation properties of our materials. For this purpose, 4-NP hydrogenation tests were conducted to assess their efficacy in driving this important reaction. The catalytic reduction of 4-NP to 4-AP was investigated to determine the catalytic activity of the SSN catalysts (Figure 4**(a) and**S20–22). UV–vis spectroscopy was used to monitor both the quantitative and qualitative development of the 4-NP reduction process. Figure 4(b) displays the UV–vis spectra for the reduction of 4-NP using SSNs. The distinctive absorption peak of 4-NP at 400 nm decreased with time, indicating that the reduction reaction had progressed. Concurrently, a new absorption peak appeared at approximately 300 nm, indicating the production of 4-AP. Furthermore, the different isosbestic points at 224, 244, 281, and 313 nm showed no side product formation.^43,44^ As shown in Figure 4(c), the conversion rates of 4-NP to 4-AP over time are plotted for the various SSN composites. The superior performance of SSN_10_ can be attributed to the increased availability of active sites, which facilitates a more efficient reduction process. The conversion rates highlight the significant impact of the Ag NPs content of the catalysts on the reaction efficiency. The reaction kinetics shown in Figure 4(d) was investigated using a first-order reaction model. The linear graphs support first-order kinetics for all catalysts, with variable slopes suggesting varied reaction rates. The highest rate constant was found to be 0.2 ± 0.0 s^–1^ for SSN_10_, followed by SSN_15_, SSN_20_, and SSN_5_, and the lowest was for SSN_0_, showing that SSN_10_ had outstanding catalytic activity for reducing 4-NP to 4-AP.

The reusability of the plasma-treated SSN nanocatalyst was further assessed through a recycling test, as shown in Figure 4(e) and Table S6. After 20 consecutive uses, the catalyst retained 86% of its conversion efficiency, thereby highlighting its potential stability and reusability. This level of performance after multiple cycles indicates that the catalyst is not only effective, but also durable, making it a promising candidate for practical applications where long-term use is required. Figure 4(f) and Table S4 compare the performance of SSN_10_ with other reported catalysts for the reduction of 4-NP to 4-AP.^45−55^ The *x*-axis represents the activity factor *k* (s^–1^ g^–1^), which quantifies the ratio of the decrease rate constant to the overall weight of the catalyst, and the *y*-axis shows the turnover frequency (TOF), indicating the catalytic efficiency in terms of the number of moles of 4-NP converted to 4-AP by 1 mg of catalyst in 1 min.^56^ SSN_10_ has an active factor k (3200 s^–1^ g^–1^) and TOF value (up to 0.096 min^–1^). Compared to other metal-based catalysts displayed on the histogram (Figure 4(f)), SSN_10_ exhibited exceptional performance, achieving a significant TOF while maintaining a substantial activity factor. SSN_10_ demonstrated a balanced combination of high TOF and activity factors, making it an efficient catalyst. Furthermore, its durability across numerous cycles indicates its potential for practical application in environmental remediation and sustainable catalysis. This establishes SSN_10_ as a competitive alternative to conventional noble metal catalysts, highlighting the importance of microplasma-assisted synthesis in the advancement of catalytic materials.

### 3.3 Dye Degradation Study of Nanocatalysts

**Figure 5:**
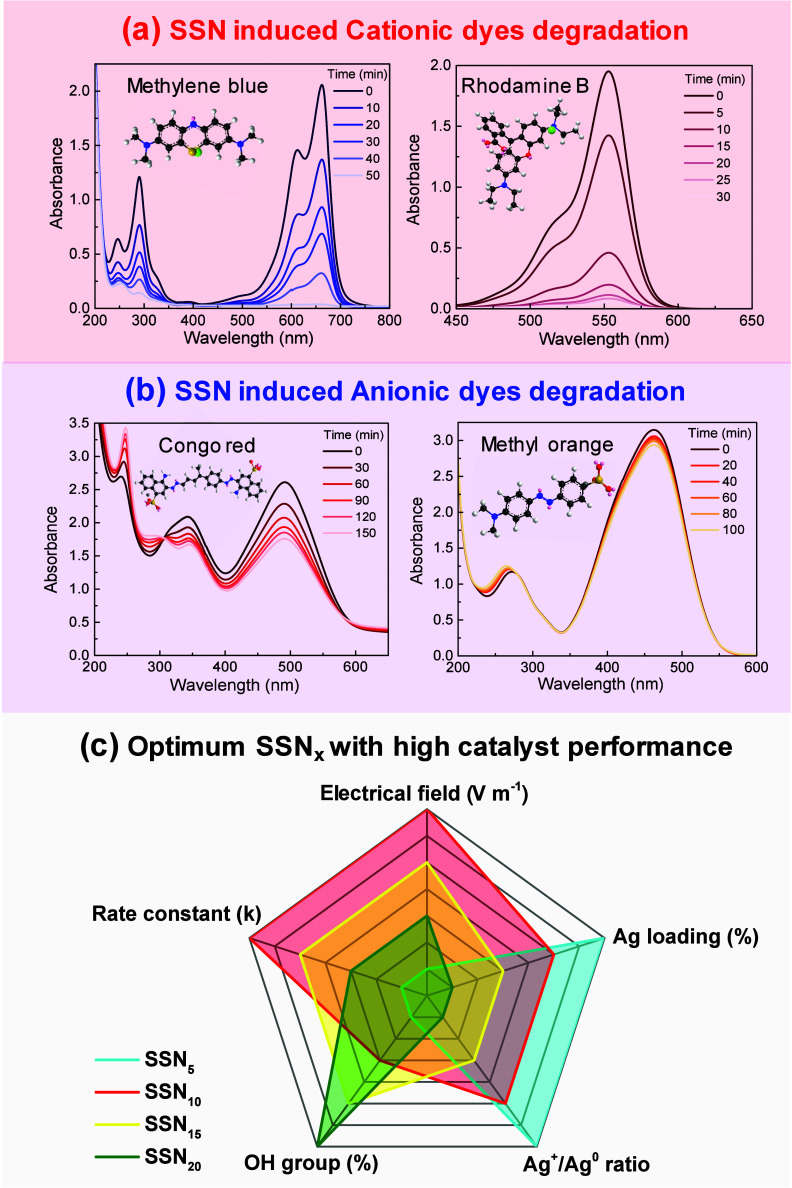
Dye degradation study of nanocatalysts. Catalytic performance of (a) cationic and (b) anionic organic dyes. (c) Comparative analysis of the catalytic properties (electrical field and rate constant of 4-NP reduction) and compositional characteristics (Ag loading, OH group percentage, and Ag^+^/Ag^0^ ratio) of self-silver nanocatalysts synthesized using various plasma treatment durations.

The catalytic activity of the SSN treated with plasma for 10 min was further evaluated by monitoring the degradation of various cationic and anionic dyes. The spectra of the cationic dyes (Figure 5(a)) exhibit a significant decrease in the characteristic absorption peaks after contact with the plasma-treated self-silver nanocatalyst. The initial absorption peaks, corresponding to the chromophore groups in the dyes, gradually diminished, indicating the disintegration of the dye molecules. The rapid reduction in the absorption intensity shows that the plasma treatment increased the catalytic activity of the SSN nanocatalysts, leading to more effective dye degradation. This observation was consistent across different cationic dyes, underscoring the broad-spectrum activity of the catalyst. The negatively charged surface of the plasma-treated SSN nanocatalyst was responsible for the higher catalytic activity observed for cationic dyes, as demonstrated by the zeta potential measurements shown in Figure S18 Following plasma treatment, the zeta potential of the catalyst was approximately −60 mV, suggesting a stable and extremely negative surface charge.^57^ This negative charge increases the electrostatic attraction between the catalyst surface and positively charged cationic dye molecules, resulting in a higher adsorption efficiency and faster degradation. In contrast, the degradation of anionic dyes (Figure 5(b)) followed a different pattern. The UV–vis spectra showed a more progressive decline in the absorption peaks than the cationic dyes. However, the degradation process was not significant, as evidenced by the substantial residual absorption at the end of the reaction. This difference in activity could be related to electrostatic interactions between the plasma-treated SSN nanocatalysts and dye molecules. The negatively charged surface of the anionic dyes may experience repulsion from the similarly charged catalyst surface, leading to a reduced adsorption efficiency and, consequently, a slower degradation rate. Despite this, the catalyst still showed some activity, indicating its potential applicability in certain scenarios, although it is more effective with cationic dyes. The selectivity of the catalyst for cationic dyes and its broad utility in degrading various pollutants, including 4-NP, make it a versatile and effective option for wastewater treatment applications where cationic contaminants are prevalent.

Figure 5(c) presents a comparative examination of SSNs generated under various conditions. A comparison of the electric field distribution among the samples shows that SSN_10_ has the strongest field, which directly correlates with its superior catalytic performance. This trend of electric field strength indicates that an optimal balance between nanoparticle size and dispersion, as seen in SSN_10_, enhances the plasmonic effect, leading to better catalytic results.^58^ SSN_10_ outperformed the other samples in terms of the catalytic performance, with the highest rate constant. This shows that the 10 min plasma treatment achieves an optimal balance of silver loading and active site creation, thereby increasing catalytic activity.^59^ In contrast, SSN_5_ and SSN_15_ exhibited modest performance, indicating that shorter and slightly longer treatments do not provide comparable advantages to the 10 min treatment. SSN_20_ had the lowest performance potential because of the longer treatment period, generating overgrowth or aggregation of silver nanoparticles and reducing the number of available active sites. SSN_5_ had the highest Ag loading, indicating a higher surface concentration of silver and shorter treatment periods. However, increasing the loading does not correlate directly with increased catalytic activity, indicating that silver dispersion and quality are important considerations. The number of OH groups increased with the treatment duration, with SSN_20_ having the greatest percentage, indicating that extended treatments may enhance the surface hydroxyl groups needed for catalytic processes. SSN_10_ utilizes a balanced number of OH groups, which potentially enhances its effectiveness by facilitating efficient adsorption and electron transfer.^60^ The Ag^+^/Ag^0^ ratio declined with treatment duration, with SSN_5_ having the highest ratio. However, the lower Ag^+^/Ag^0^ ratio in SSN_20_ results in only modest catalytic activity, suggesting that an ideal ratio, rather than the greatest, is crucial for improved performance.^61^ SSN_10_ produces a balanced Ag^+^/Ag^0^ ratio, which improves electron transport processes and catalytic efficiency. The 10 min plasma treatment (SSN_10_) achieved optimum catalytic characteristics by balancing the Ag loading, surface OH groups, and Ag^+^/Ag^0^ ratio. Tuning the synthesis parameters correctly is critical for increasing the catalytic efficacy of self-Ag nanocatalysts.

## 4 Conclusion

This study presents a plasma engineering approach for recycling PC optical discs into high-performance nanocarbon-supported Ag nanocatalysts using a simple and controllable atmospheric-pressure microplasmas. The plasma-engineered nanocatalysts demonstrated exceptional catalytic activity, particularly in the rapid reduction of the hazardous pollutant 4-NP to the valuable intermediate 4-AP, within an unprecedented 30 s, and exhibited potential durability, maintaining up to 86% conversion efficiency over 20 consecutive cycles. Precise tuning of the plasma exposure time was identified as a critical factor in optimizing the catalytic properties of nanocatalysts. The 10 min plasma treatment optimally balanced Ag loading, surface hydroxyl groups, and the Ag^+^/Ag^0^ ratio, enhancing electron transport, active site accessibility, and catalytic performance. This enabled strong electrostatic interactions with cationic dyes, demonstrating the versatility of SSN nanocatalysts for pollutant remediation. Our study provides the way for an effective synthesis and applications of nanocarbon–metal materials, offering a compelling strategy for transforming plastics waste into high-value functional materials that can contribute to a cleaner and more sustainable future.

## Supporting Information Available

Experimental setup and additional analysis including XPS, XRD, AFM, SEM, TEM, UV–vis absorption, Raman, contact angle and zeta potential measurements (PDF)
