# Supplementary material for: Compact Disc-Derived Nanocarbon-Supported Catalysts with Extreme Catalytic Activity
Source: ACS Appl Mater Interfaces. 2025 Jan 22;17(5):8147–57. doi: 10.1021/acsami.4c17754 (PMC11803566; doi:10.1021/acsami.4c17754)
Supplement: Supplementary file 1 — am4c17754_si_001.pdf [file am4c17754_si_001.pdf]

Supporting Information

Compact Disc-Derived Nanocarbon-Supported Catalysts with Extreme Catalytic Activity

Chia-Hung Lin[1], *Yi-Jui Yeh*[1,2], *Tzu-Hsiang Chien*[1], *Shao-Yu Chen*[2], *Loganathan Veeramuthu*[3], *Chi-Ching Kuo*[3,4], *Kuo-Lun Tung*[2] *and Wei-Hung Chiang*[1,5]*

[1]Department of Chemical Engineering, National Taiwan University of Science and Technology, Taipei 10607 Taiwan

[2]Department of Chemical Engineering, National Taiwan University, Taipei, 10607 Taiwan

[3]Institute of Organic and Polymeric Materials, Research and Development Center of Smart Textile Technology, National Taipei University of Technology, Taipei 10608, Taiwan

[4]Advanced Research Center for Green Materials Science and Technology, National Taiwan University, Taipei 10607, Taiwan

[5]Sustainable Electrochemical Energy Development (SEED) Center, National Taiwan University of Science and Technology, Taipei City 10607, Taiwan

*Corresponding author: E-mail address: *whchiang@mail.ntust.edu.tw* (W.H.C)

This supplementary information file contains 44 pages

S1. Materials and chemicals

Silver-carbon plates were obtained from waste polycarbonate discs or local companies. Sodium hydroxide (NaOH, ≥98%, CAS: 1310-73-2) pellet, Malachite Green chloride (MG, ≥99%, CAS: 569-64-2), Rhodamine B (RB, ~95%, CAS: 81-88-9) were purchased from Sigma Aldrich Co., Ltd. Methylene blue (MB, CAS: 122965-43-9), Congo red (CR, CAS: 573-58-0) and 4-nitrophenol (4-NP, 99%, CAS: 100-02-7) were obtained from Alfa Aesar. Sodium borohydride ($NaBH_4$, 98+%, CAS: 16940-66-2) were received from Acros Organics. PTFE reactor (inner volume: 50 × 40 × 10 mm, thickness 4 mm).

S2. Materials characterizations

Absorbance spectra of all samples were collected using a JASCO V676 absorbance spectrophotometer and a matched set of 1 cm path length quartz cuvettes. DI water were used as a baseline reference for every spectral measurements. Raman measurements were performed using a JASCO-5100 confocal Raman spectrometer with laser excitation wavelengths green (532.17 nm). The spectral resolution was 0.5-1.0 cm^{-1}, and the wave-number range for the measurement is from 1000-1800 cm^{-1}. The chemical compositions were performed by Fourier-transform infrared (FTIR) transmittance spectroscopy (SHIMADZU Tracer-100), with attenuated total reflection (ATR) mode. X-ray photoelectron spectroscopy (XPS, ULVAC-PHI. Inc., PHI 5000 VersaProbe Ⅲ) were conducted to determine com-position and elemental analysis. Scanning electron microscope (SEM) measurement was performed using a field emission SEM (JEOL JSM-IT800) with an EDX detector. Transmission electron micro-scope (TEM) measurements were performed using a field emission gun TEM (FEI Tecnai™ G2 F-20 S-TWIN) with an accelerating voltage of 200 kV. As-prepared products were divided into 1 × 1 cm^2 and sonicated in 5 mL ethanol for 20 mins before being drop-cast on copper grids covered with formvar/carbon. (300 mesh, Ted Pella, Inc.). TGA (NETZSCH STA 449 F3 Jupiter) measurements were conducted with nitrogen gas flow, with a temperature range of 100 to 800 °C and a heating rate set to 10 °C per minute. The crystal structures of samples were obtained employing an X-ray diffractometer (XRD, D2 PHASER XE-T X-ray Powder Diffractometer) by Cu-Kα radiation under 30 kV and 10 mA. The surface morphology were analyzed by atomic force microscopy (AFM, Bruker Dimension ICON). Zeta potential measurements were performed by solid surface zeta potential analyzer (Anton Paar SurPASS 3). Contact angle measurements were conducted using an optical contact angle meter (KINO SL150E).

S3. Nanocarbon-support study of Self-Silver Nanocatalysts

The TEM image of nanocarbon-support of self-silver nanocatalyst provides in **Figure S3**. Inverse FFT image, highlights distinct lattice fringes with spacings of 0.231 and 0.225 nm, corresponding to the (100) and (1120) planes of graphite and graphene [1, 2]. These findings validate the crystallinity and specific lattice orientations of the synthesized graphene dots as nanocarbon-support.

Based on the Raman spectra presented in **Figure S23**, only the characteristic D- and G-bands associated with graphene structures are observed, with the G-band showing a relatively higher intensity than the D-band. The G-band is indicative of the crystallinity and graphitic nature of the nanocarbon material due to the presence of C=C bonds, while the D-band reflects structural defects and functional groups. The I_D/I_G ratios, in ascending order, are SSN_{10}, SSN_{15}, SSN_{20}, and SSN_5. This trend correlates with the degradation efficiency of 4-NP, where samples with a higher G-band intensity, such as SSN_{10}, exhibit higher crystallinity. The increased crystallinity suggests stronger electron conductivity, which enhances the catalytic performance for 4-NP reduction [3]. This result confirms the presence of nanocarbon support on the substrate, with high purity and a well-ordered crystalline structure, contributing to the overall catalytic effectiveness.

TEM and Raman analyses confirm the presence of high-purity nanocarbon support in the self-silver nanocatalyst, with the I_D/I_G ratios correlating higher crystallinity to improved electron conductivity and enhanced catalytic performance for 4-NP reduction.

S4. Electric field distribution study of Self-Silver Nanocatalysts

The electric field distribution of the Ag-based material was simulated using COMSOL Multiphysics, employing the wave optics module to model the localized surface plasmon resonance (LSPR) effects around a silver nanoparticle. A 2D geometry was constructed to represent the nanoparticle in a homogeneous dielectric environment, with the particle size and shape derived from experimental data (**Figure S14**) corresponding to the Ag nanoparticles synthesized under specific plasma conditions. The model utilized the finite element method (FEM) to solve Maxwell's equations for the electric field, with a perfectly matched layer (PML) applied at the outer boundaries to prevent spurious reflections. The material properties of silver, including the complex refractive index, were obtained from standard optical data for Ag in the visible spectrum. The surrounding medium was assumed to have a refractive index of 1.3782 with a small imaginary component to account for absorption [4].

S5. Mechanism study of hot electron transfer via Surface-enhanced Raman spectroscopy

The Raman spectra (**Figure S15**) provide evidence supporting the involvement of hot electron transfer in the catalytic mechanism of SSN_{10}. The characteristic peaks of 4-NP, appearing at around 863, 1105, 1212, 1322, and 1586 cm^{-1}, are significantly enhanced in the presence of SSN_{10}, as compared to others [5]. This enhancement is associated to the localized surface plasmon resonance (LSPR) effect [6], which interact with adsorbed 4-NP molecules to drive the catalytic reaction. Although LSPR-induced hot electron transfer was proposed as a potential factor in the catalytic reduction of 4-NP, the experiment was primarily conducted to confirm the original proposition. These findings support the proposed mechanism while acknowledging the potential presence of other LSPR-related effects such as localized heating.

S6. Surface Functional Groups Analysis of Self-Silver Nanocatalysts

FTIR spectra analysis (**Table S2**) shows clear differences in the surface functional groups before and after plasma treatment. The untreated polycarbonate (PC) disc reveals the presence of aromatic and alkyl groups via C=C bending vibrations at 1566 cm^{-1} and C-H bending vibrations at 1448 and 1413 cm^{-1}. Additionally, C-O stretching is observed at 1232 and 1195 cm^{-1}, indicating ester or ether functionalities.

After undergoing atmospheric-pressure microplasma treatment, the self-silver nanocatalyst (SSN) exhibits notable changes. Significant alterations in surface functional groups were detected following plasma treatment, especially with the addition of hydroxyl (-OH) and carbonyl (C=O) groups. The increase in oxygen-containing functional groups, facilitated by NaOH treatment, further supported the plasma-induced reduction process, enabling the formation of a greater amount of metallic state silver. This increase in metallic silver significantly contributed to the enhanced catalytic performance of the SSN.

The appearance of OH stretching at 3400 cm^{-1} suggests surface hydroxylation, while CO_2 stretching vibrations at 2345 cm^{-1} point to possible surface adsorption. The formation of carbonyl-containing species, such as aldehydes or ketones, is evident through C=O stretching at 1728 cm^{-1}. The presence of C=C stretching vibrations at 1651 and 1585 cm^{-1} indicates that some aromatic character remains, while C-H bending at 1365 cm^{-1} suggests retained alkyl groups. The observed C-O stretching at 1122 and 1245 cm^{-1} implies the formation of oxygen-containing groups post-treatment, possibly due to surface oxidation processes.

These changes suggest the introduction of new functional groups, particularly oxygenated species, which are likely responsible for the enhanced catalytic activity of the SSN.

S7. Surface Chemical Composition and Wettability Analysis of Self-Silver Nanocatalysts

Table 1 summarizes the surface chemical species and wettability properties of SSNs synthesized at varying plasma reaction times (5, 10, 15, and 20 minutes), labeled as SSN_5, SSN_{10}, SSN_{15}, and SSN_{20}, respectively. The surface chemical species were characterized using X-ray photoelectron spectroscopy (XPS), focusing on the Ag 3d and O 1s regions.

For the Ag 3d spectra, the binding energies (B.E.) of 367.3 eV and 368.3 eV correspond to Ag^+ and metallic Ag^0 species, respectively. The content percentages of these species reveal a decreasing Ag^+/Ag^0 ratio with increased plasma reaction time, indicating a progressive reduction of Ag^+ to Ag^0.

The O 1s spectra reveal surface oxygen species (**Figure S17**), including Ag_2O (529.2 eV), hydroxyl groups (C–OH, 530.9 eV), carboxyl groups (C=O, COOH, 532.1 eV), and ether groups (C–O–C, 533.2 eV). The relative content of these species reflects the evolving surface chemistry with prolonged plasma exposure, showing an increase in hydroxyl and carboxyl functionalities, which play a crucial role in enhancing the catalytic performance and stability of the SSNs.

The contact angle measurements highlight the wettability changes of the SSN surfaces, decreasing from 90.5° for SSN_5 to 22.8° for SSN_{20}, indicating a transition from hydrophobic to highly hydrophilic surfaces. This trend corresponds to the increased surface oxidation and the enrichment of hydroxyl and carboxyl groups, which improve water affinity and are beneficial for catalytic applications involving aqueous environments.

These findings collectively illustrate the impact of plasma reaction time on the surface chemical composition, oxidation state, and wettability of SSNs, providing valuable insights into tailoring surface properties for enhanced catalytic and environmental performance.

S8. Economic estimation for plasma-engineered self-silver nanocatalyst

The affordability of a technology is a crucial factor for its widespread application. In the case of self-silver nanocatalysts (SSNs), the primary cost is the use of sodium hydroxide (NaOH) purchased from Sigma Aldrich at $23.03/kg, while the other components are derived from recycled polycarbonate optical discs, making the process highly economical. Given the simplicity of the materials required and the reasonable cost of NaOH, the overall expense for SSN production remains within an acceptable range. Future research could focus on recycling different types of plastic waste to further develop this as a versatile and sustainable recycling approach.

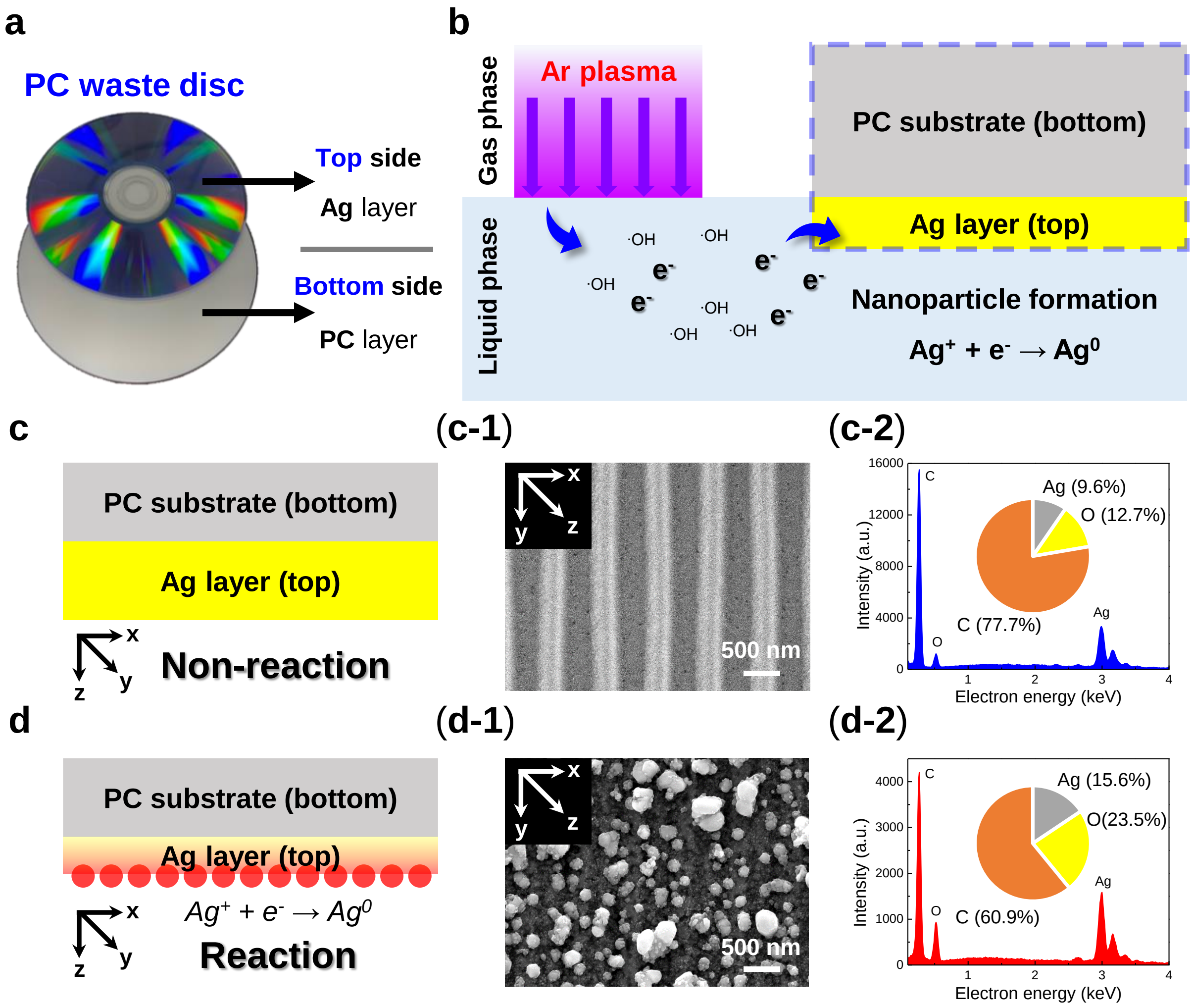

Figure S1. Schematic illustration and characterization of plasma-treated PC waste discs. (a) Structure of the PC waste disc, consisting of a top Ag layer and a bottom PC substrate layer. (b) Schematic of the microplasma treatment process, demonstrating nanoparticle formation through the reduction of Ag^+ to Ag^0 facilitated by Ar plasma in the liquid phase. (c) Non-reacted state of the PC waste disc: (c-1) SEM image showing the smooth Ag layer surface and (c-2) corresponding EDX analysis confirming the elemental composition. (d) Reacted state of the PC waste disc after plasma treatment: (d-1) SEM image showing the formation of Ag nanoparticles on the surface and (d-2) corresponding EDX analysis confirming the presence of Ag nanoparticles.

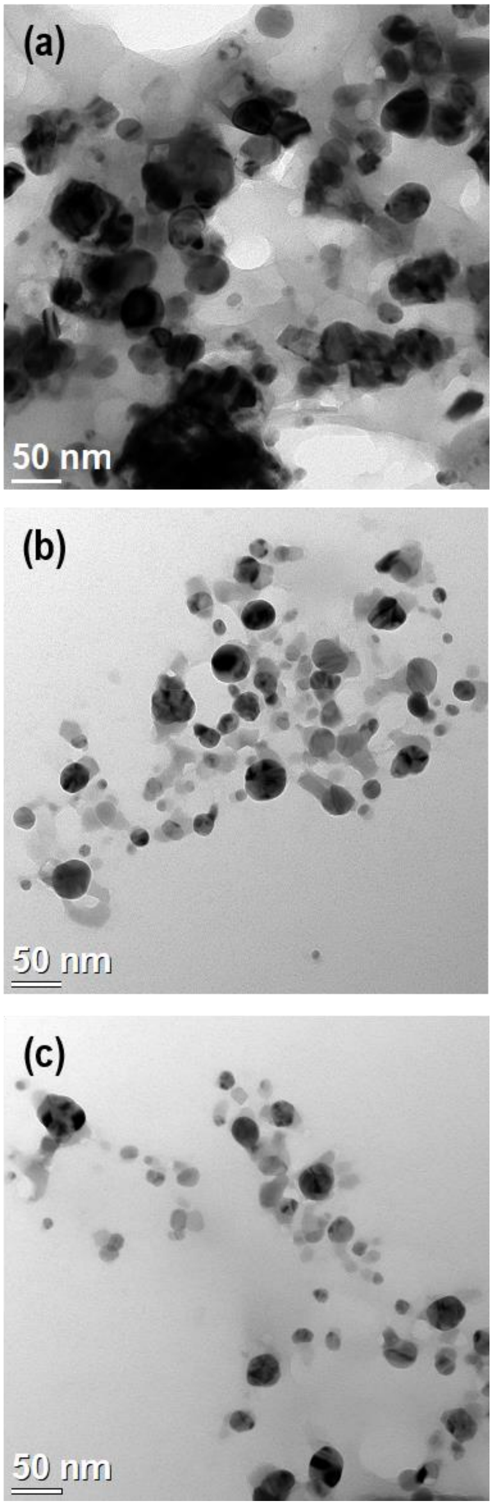

Figure S2. Transmission electron microscopy images of SSN_x (a) 5, (b) 15 and (c) 20.

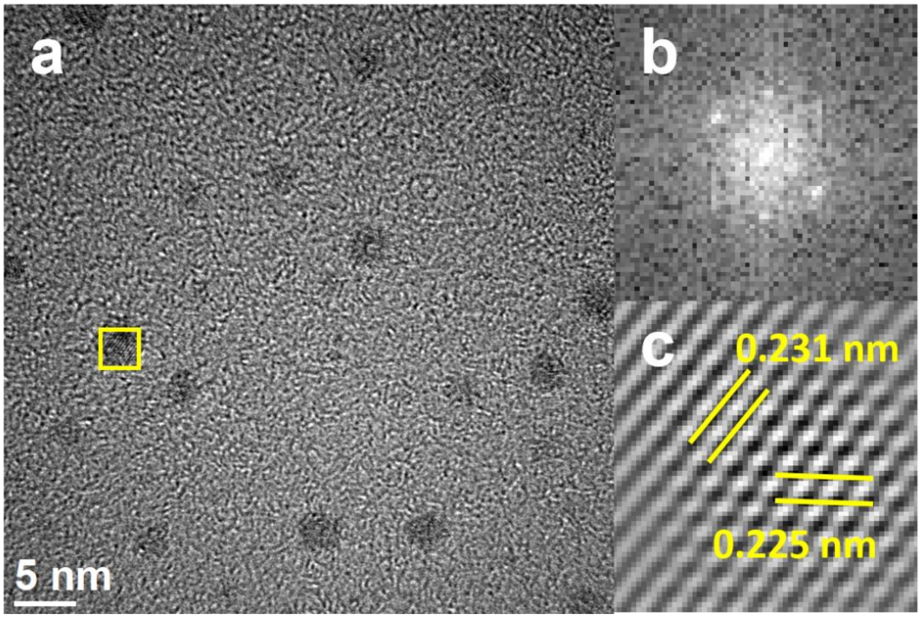

Figure S3. Transmission electron microscopy of nanocarbon-support (a) TEM image, (b) corresponding diffraction patten and (c) inverse FFT image.

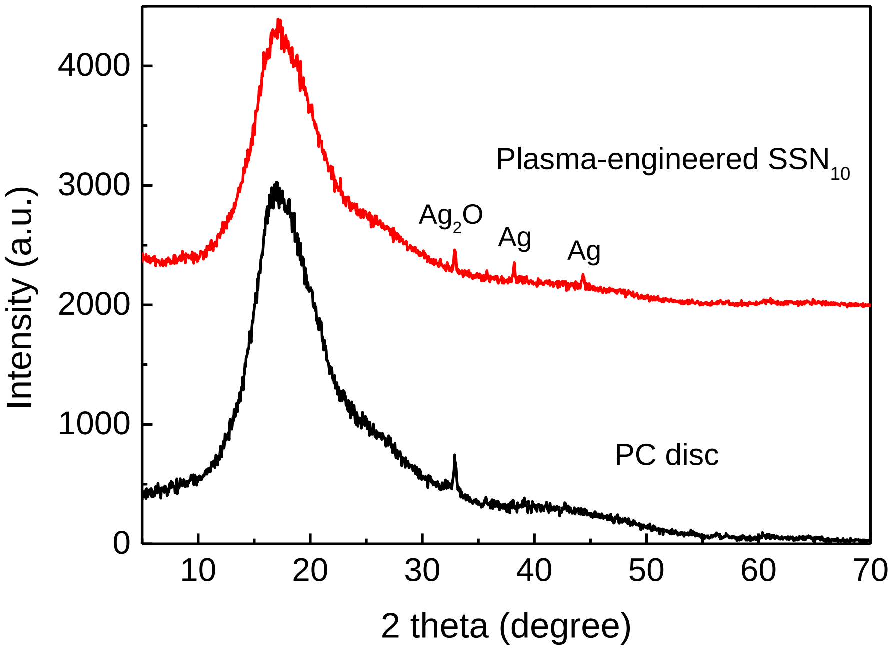

Figure S4. X-ray diffraction pattern of the synthesized SSN and PC disc.

Note.

The XRD patterns in the figure compare the untreated polycarbonate (PC) disc and plasma-engineered SSN_{10}. The plasma-engineered SSN_{10} sample exhibited diffraction peaks at 38.2° and 44.3°, corresponding to the (111) and (200) planes of crystalline silver (Ag), respectively [7]. A weak peak at 32.8° indicates the presence of residual silver oxide (Ag_2O), which is diminished after plasma treatment, confirming the reduction of silver oxide to metallic silver nanoparticles. This highlights the efficacy of plasma treatment in transforming the PC substrate into a crystalline silver-based nanocatalyst.

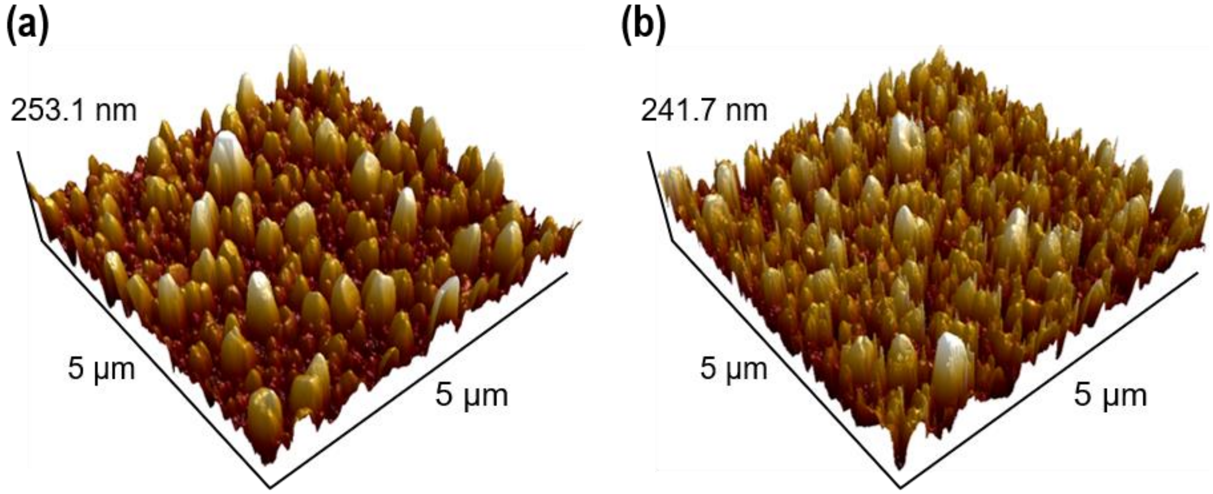

Figure S5. AFM images of (a) SSN_5, (b) SSN_{15}.

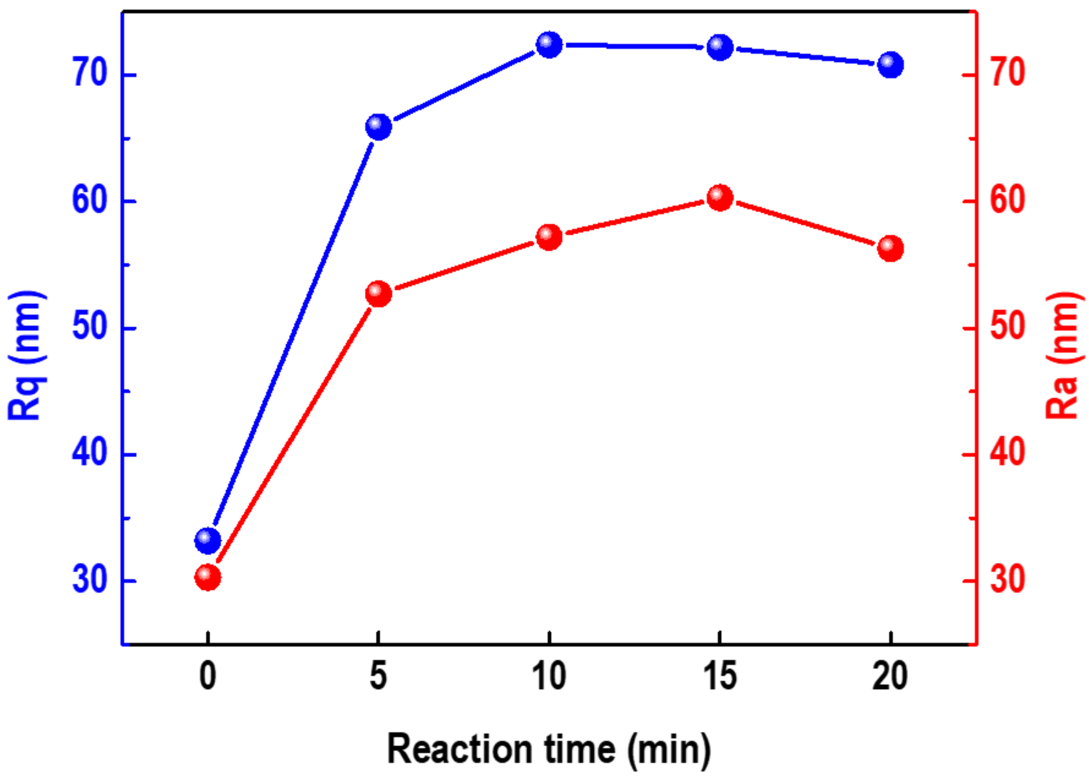

Figure S6. Surface roughness analysis of synthesized SSNs.

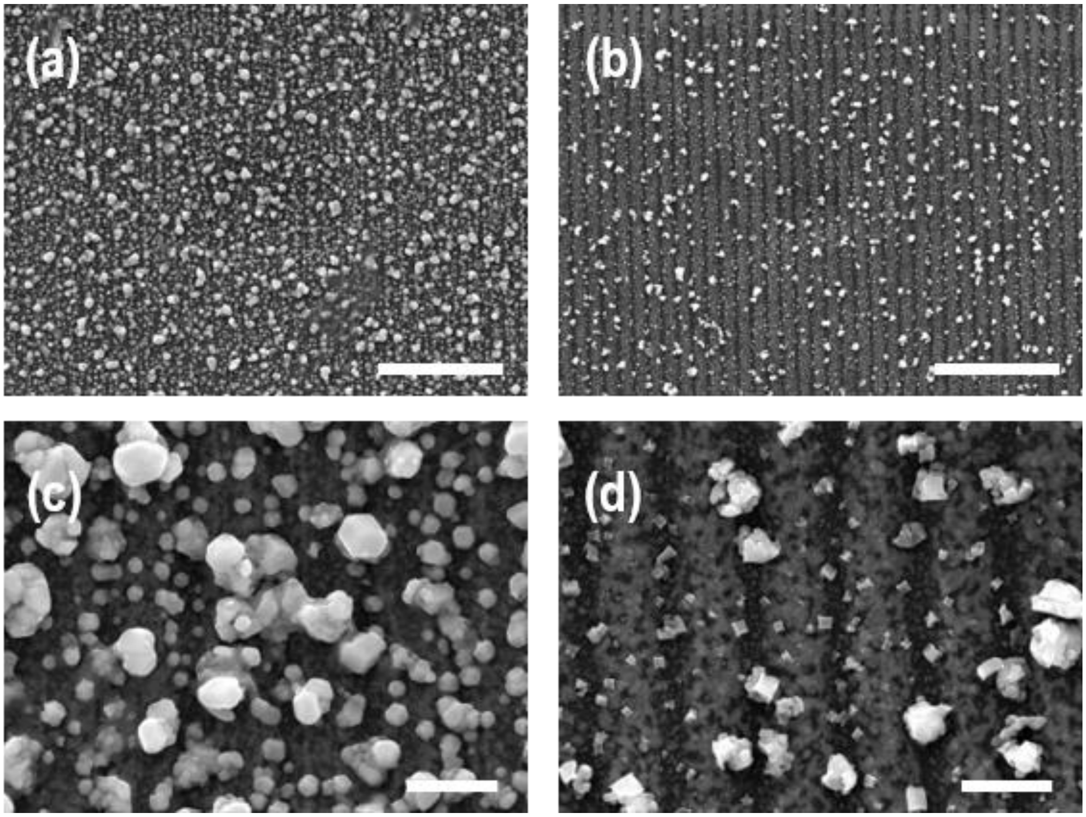

Figure S7. SEM images of (a) SSN_5, (b) SSN_{15}. (Scale bar is 5 μm). High-magnification of (c) SSN_5, (d) SSN_{15}. (Scale bar is 500 nm).

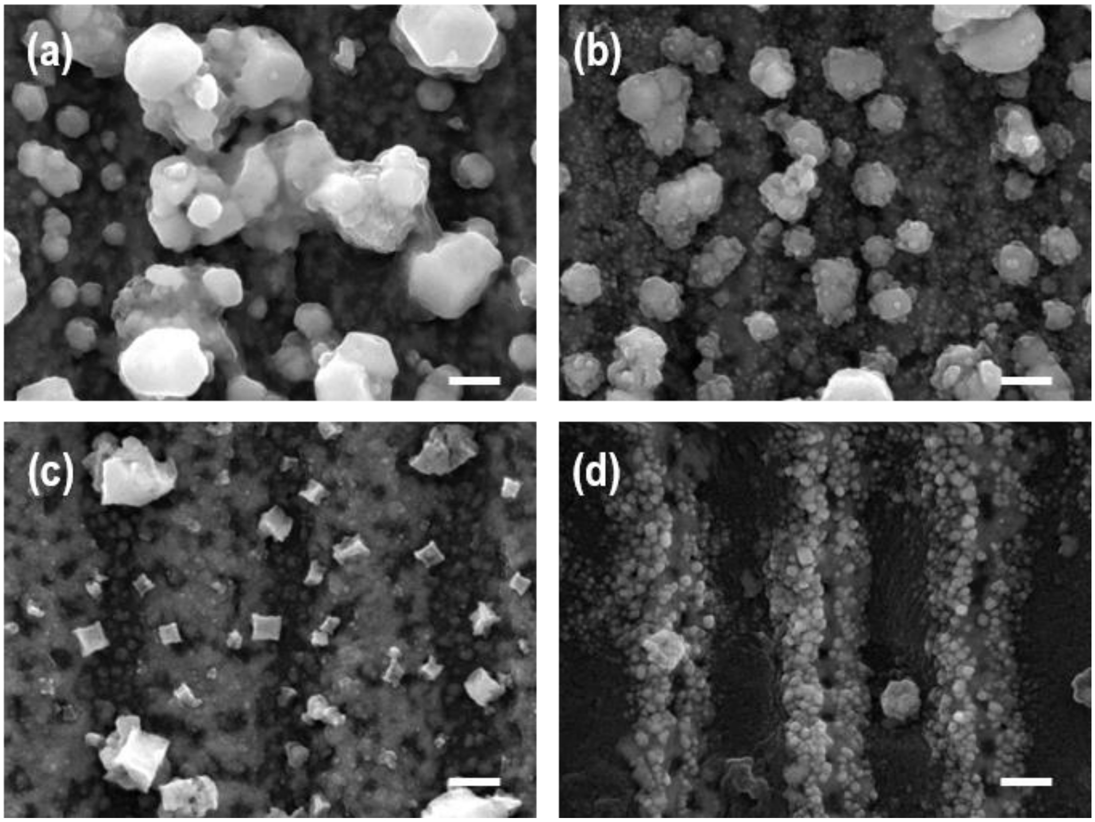

Figure S8. High-resolution SEM images of (a) SSN_5, (b) SSN_{10}, (c) SSN_{15}, and (d) SSN_{20}. (Scale bar is 200 nm).

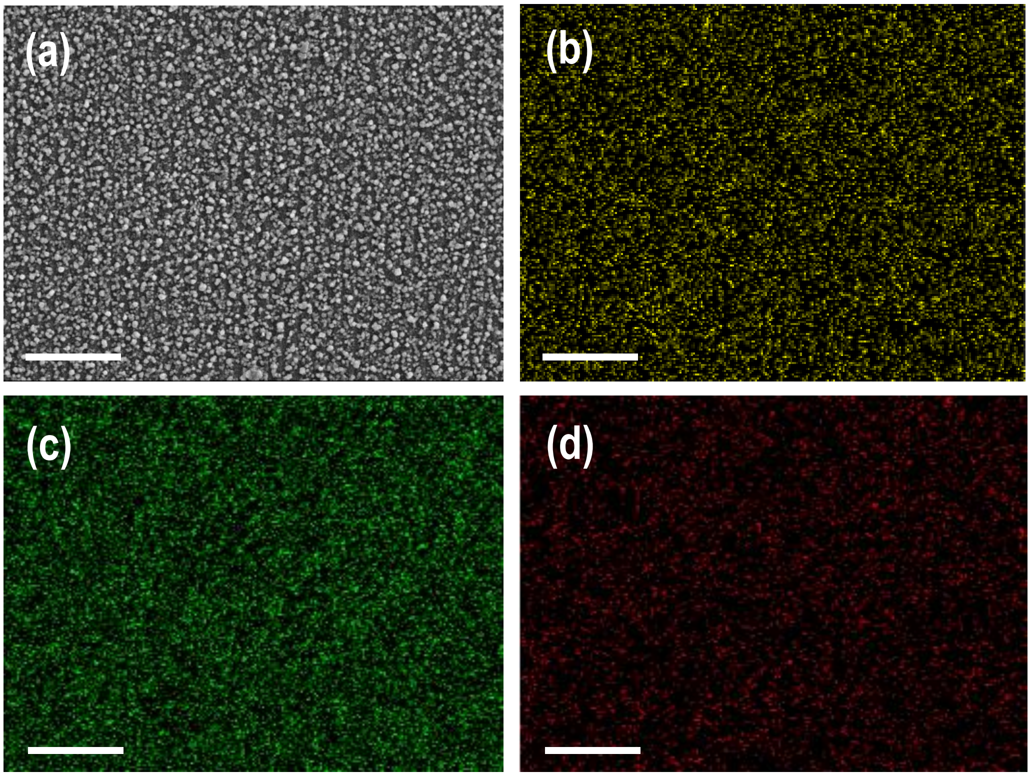

Figure S9. (a) Surface morphology of SSN_{10} by SEM and EDX analysis of (b) Ag, (c) C and (d) O mapping. (Scale bar is 5 μm).

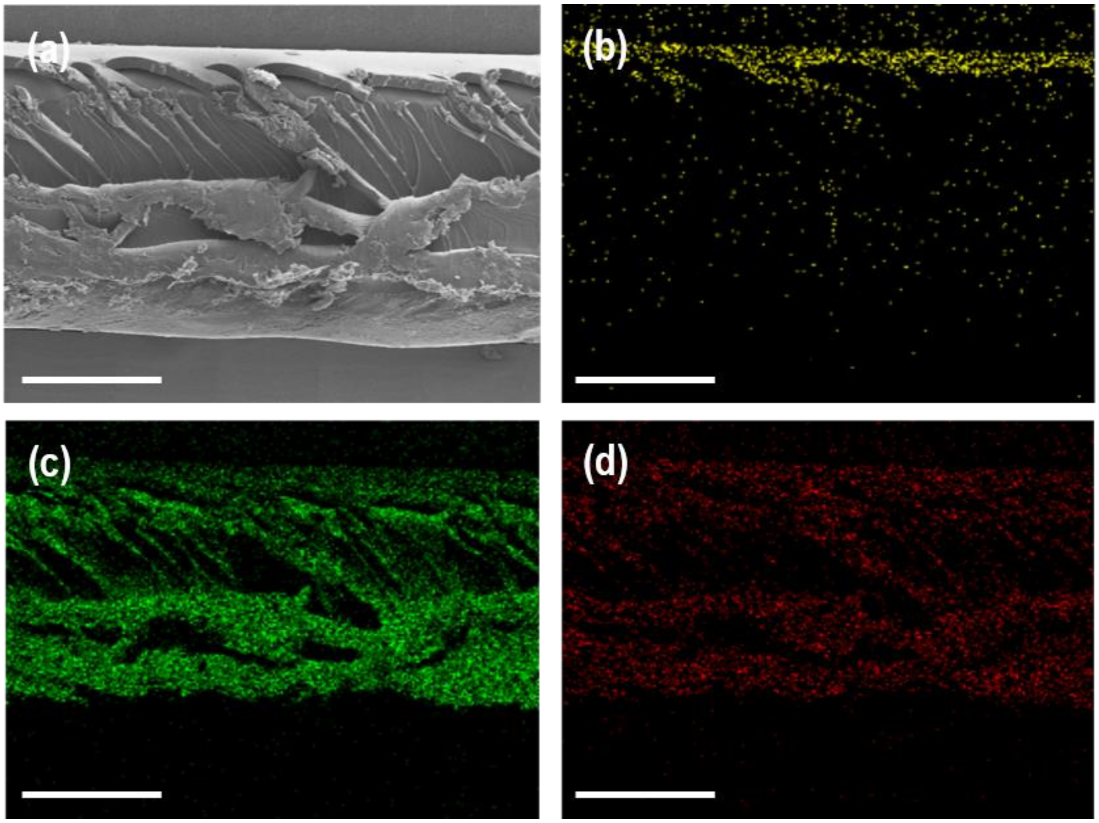

Figure S10. (a) Cross-section image of SSN_{10} by SEM and EDX analysis of (b) Ag, (c) C and (d) O mapping. (Scale bar is 250 μm).

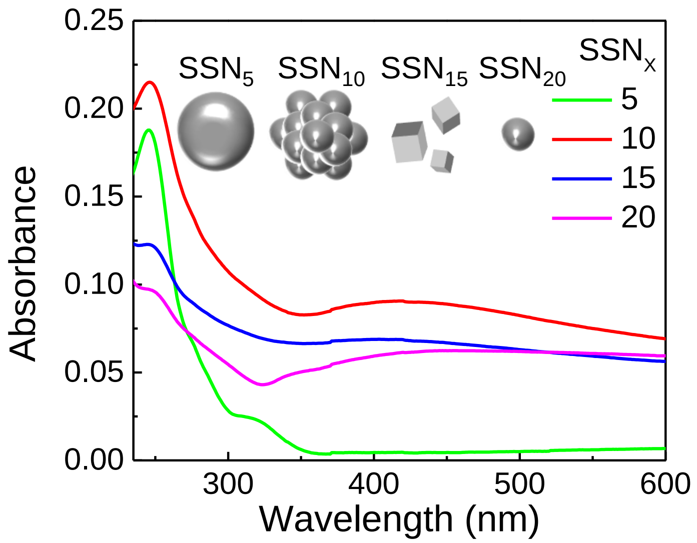

Figure S11. Absorption spectra of different synthesized time SSNs.

Note.

The absorption peak around 420 nm corresponds to the LSPR of Ag nanoparticles [8]. The intensity and position of the SPR peak varied with reaction time, suggesting changes in the particle size and distribution as the reaction progressed. Additionally, the solution contains nanocarbon, which facilitates charge transfer between the Ag NPs and nanocarbon, leading to further broadening of the SPR band [9].

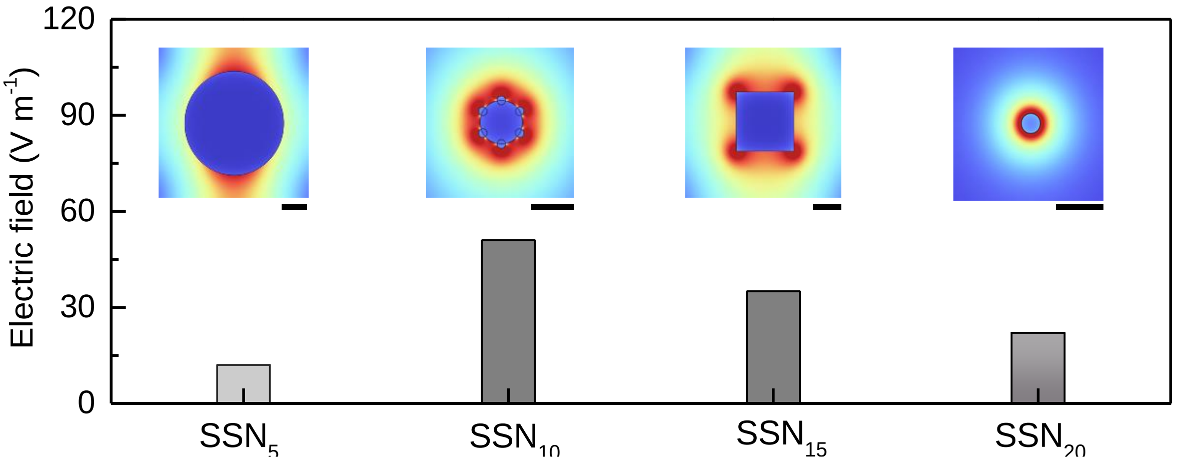

Figure S12. Simulated electric field distribution. (Scale bar: 50 nm).

Note.

Notably, SSN_{10} demonstrated the most pronounced electric field enhancement, as evidenced by the highly localized and intense red regions surrounding the nanoparticles. This significant enhancement signifies a strong LSPR effect, which is driven by the optimized morphology and particle size distribution uniquely achieved under these synthesis conditions. The intensified electric field reflects a higher density of surface charges, effectively amplifying the LSPR response, and consequently, the plasmonic properties of the nanoparticles. The robust LSPR observed in SSN_{10} not only improves the optical absorption, but also facilitates electron excitation, significantly enhancing the catalytic activity. The hot electrons generated from LSPR can be transferred to adjacent molecules, lowering the activation energy of the catalytic reactions and accelerating the reaction rates. This electron transfer mechanism is a potential factor for the superior catalytic performance of SSN_{10}.

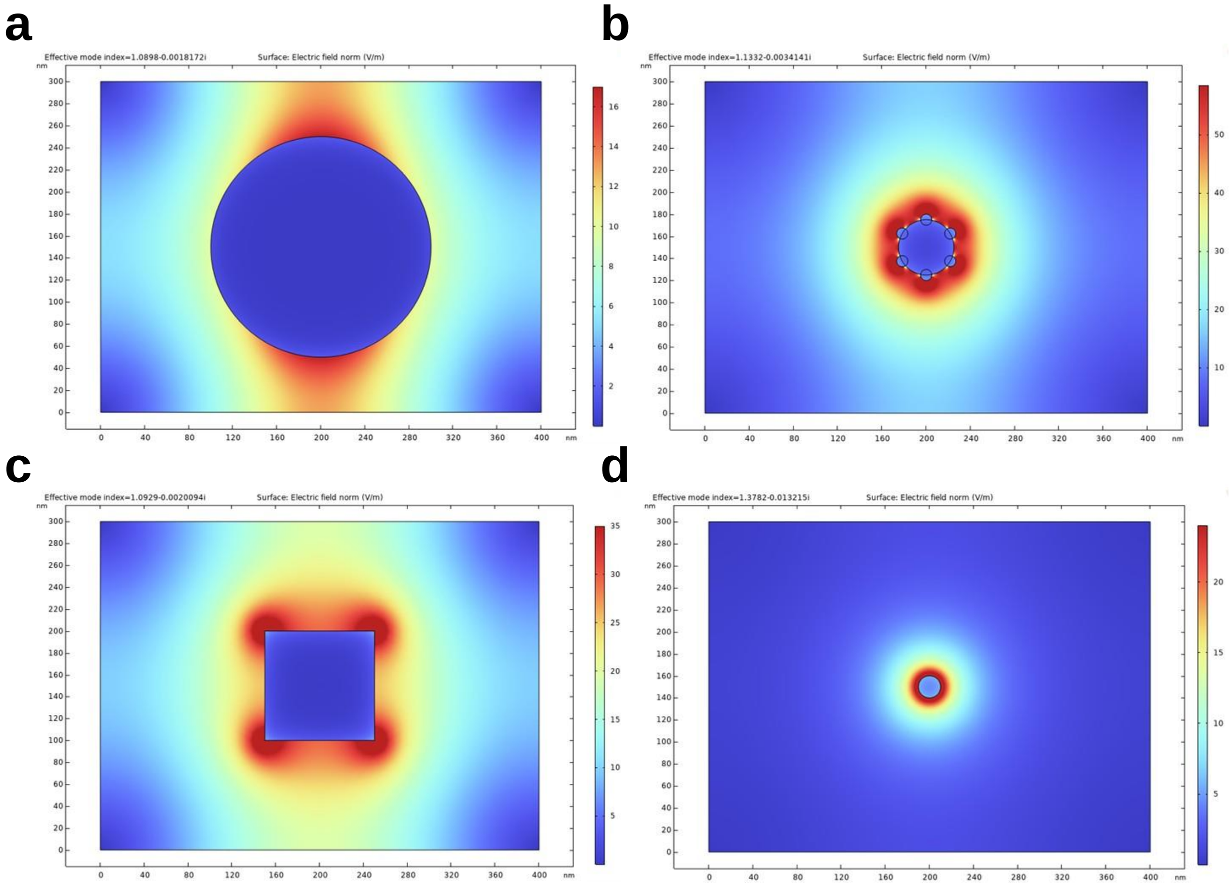

Figure S13. Electric field distribution of different synthesized time SSNs (a) 5, (b) 10, (c) 15 and (d) 20.

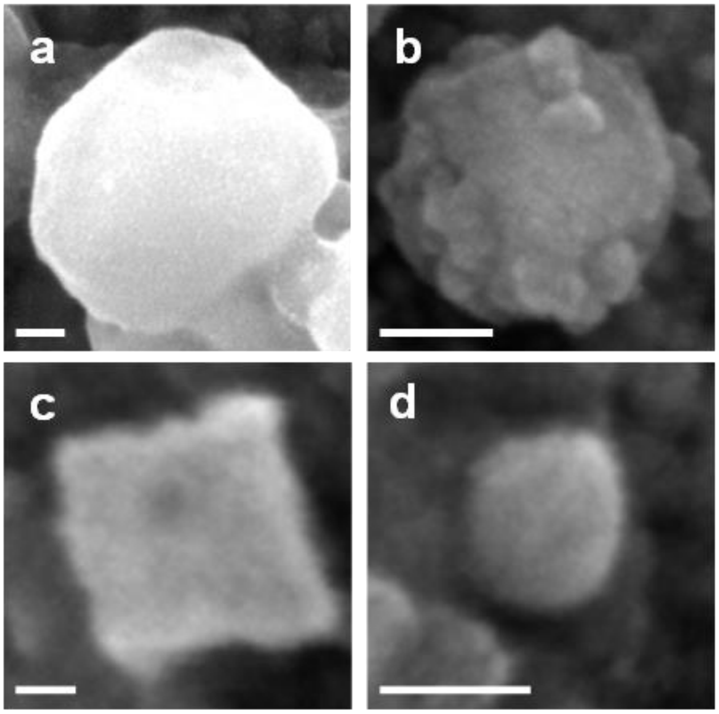

Figure S14. SEM images of the model supporting (a) SSN_5, (b) SSN_{10}, (c) SSN_{15} and (d) SSN_{20}. (Scale bar: 25 nm)

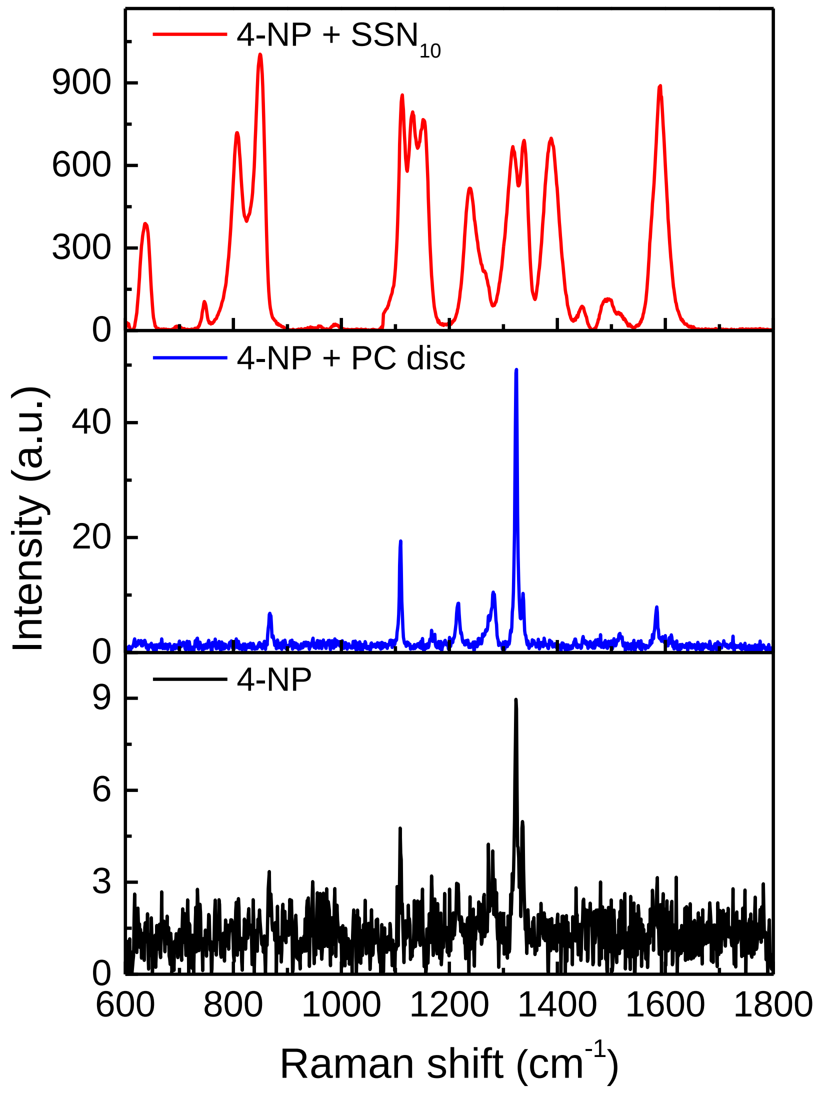

Figure S15. Surface-enhanced Raman spectroscopy for hot electron transfer study.

Raman spectra demonstrating the enhancement of 4-NP signals under different conditions: (top, red) 4-NP in the presence of SSN_{10}, showing significant enhancement with characteristic peaks at ~863, 1105, 1212, 1322, and 1586 cm^{-1} due to the LSPR effect; (middle, blue) 4-NP with the untreated PC disc, displaying moderate enhancement; and (bottom, black) 4-NP without any substrate, showing minimal signal intensity. These results highlight the superior catalytic performance of SSN_{10} in facilitating SERS.

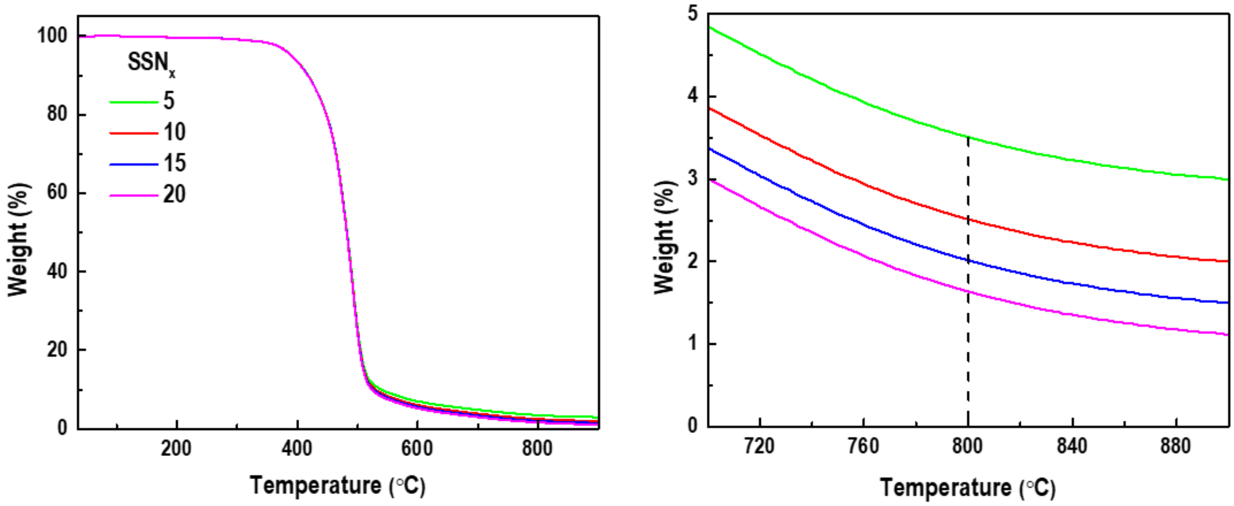

Figure S16. TGA spectra of synthesized SSNs under different plasma treatment times.

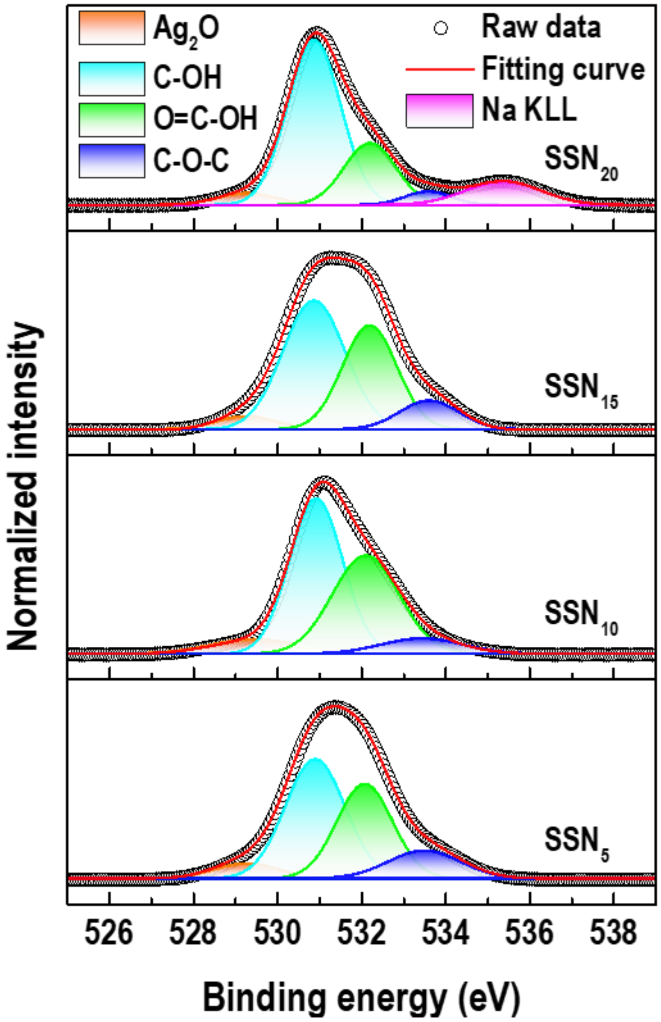

Figure S17. HRXPS measurement of O 1s.

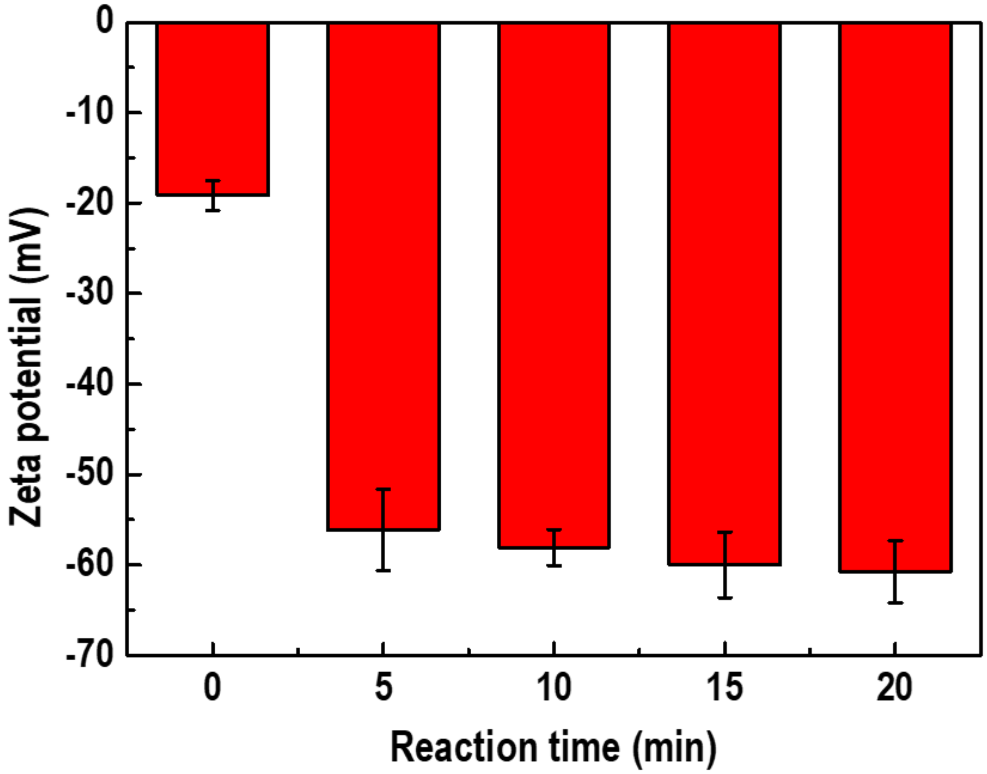

Figure S18. Zeta potential of synthesized SSNs under different plasma treatment times.

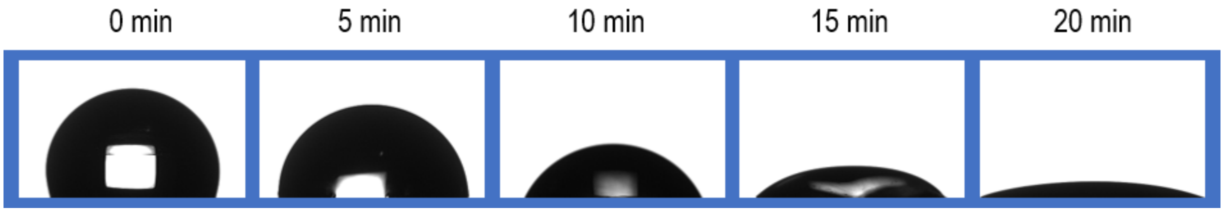

Figure S19. Contact angle photography of synthesized SSNs.

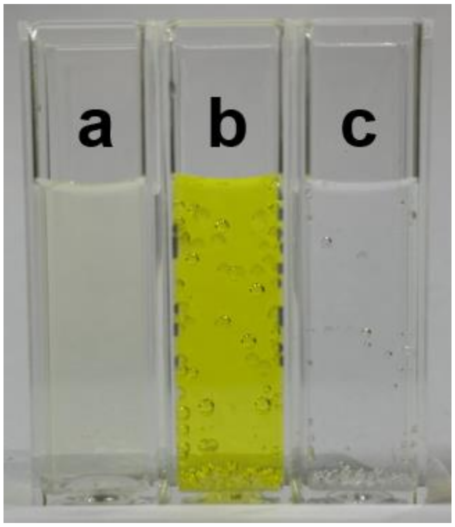

Figure S20. Photographs of (a) starting 4-NP solution before catalysis reduction, (b) after adding $NaBH_4$ solution, (c) 4-AP solution after catalysis reduction.

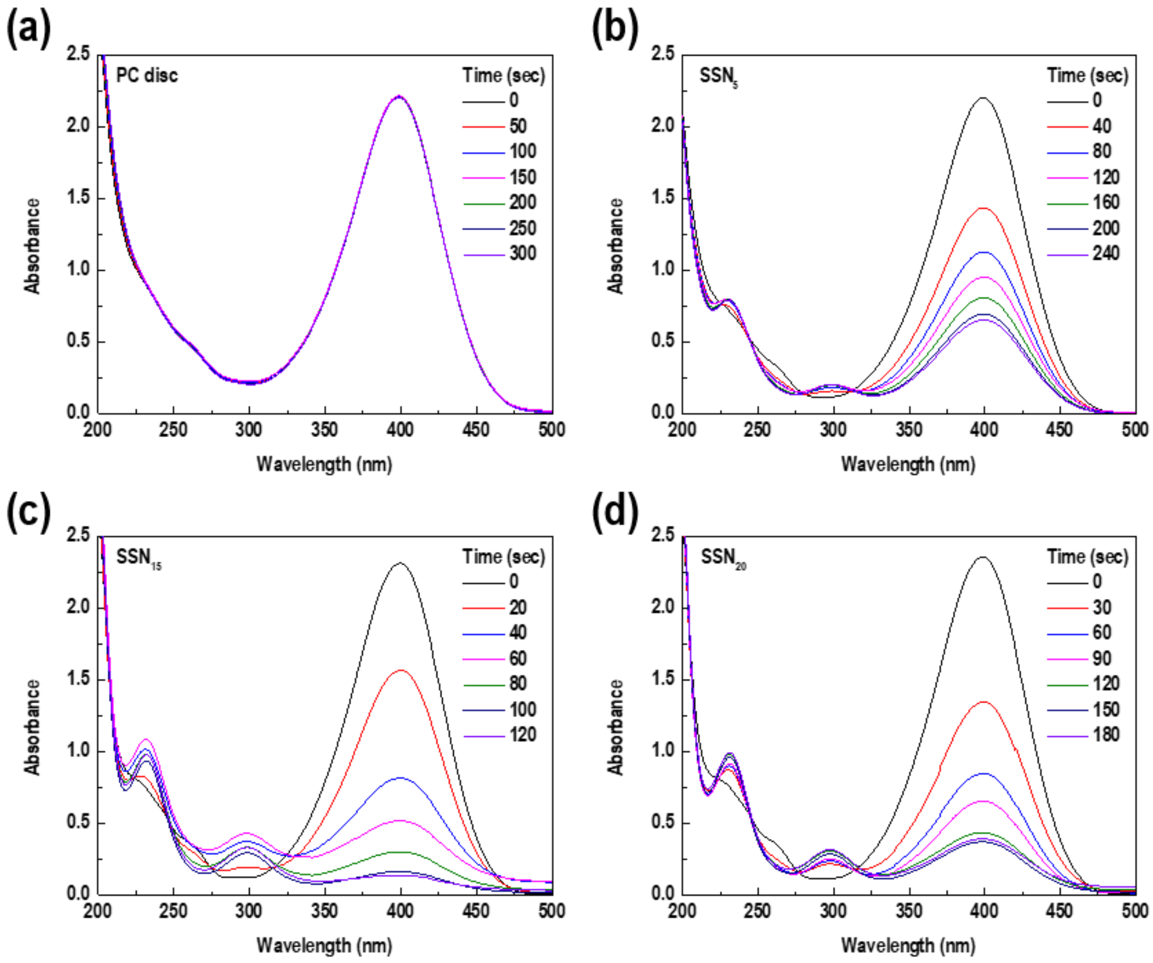

Figure S21. UV-vis spectra of 4-NP after catalytic degradation using SSN_x under different plasma treatment times. (a) 0, (b) 5, (c) 15 and (d) 20 min.

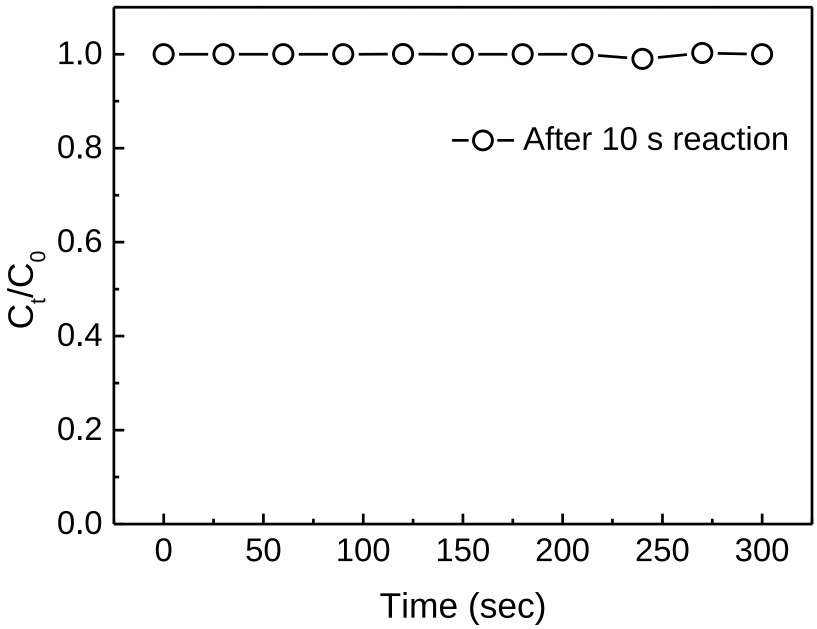

Figure S22. Reduction quenching process during the SSN extraction

Note

This figure demonstrates the stability of the reaction after quenching during the SSN extraction. The result confirms that the reaction was effectively halted immediately upon extraction, with no further progress observed.

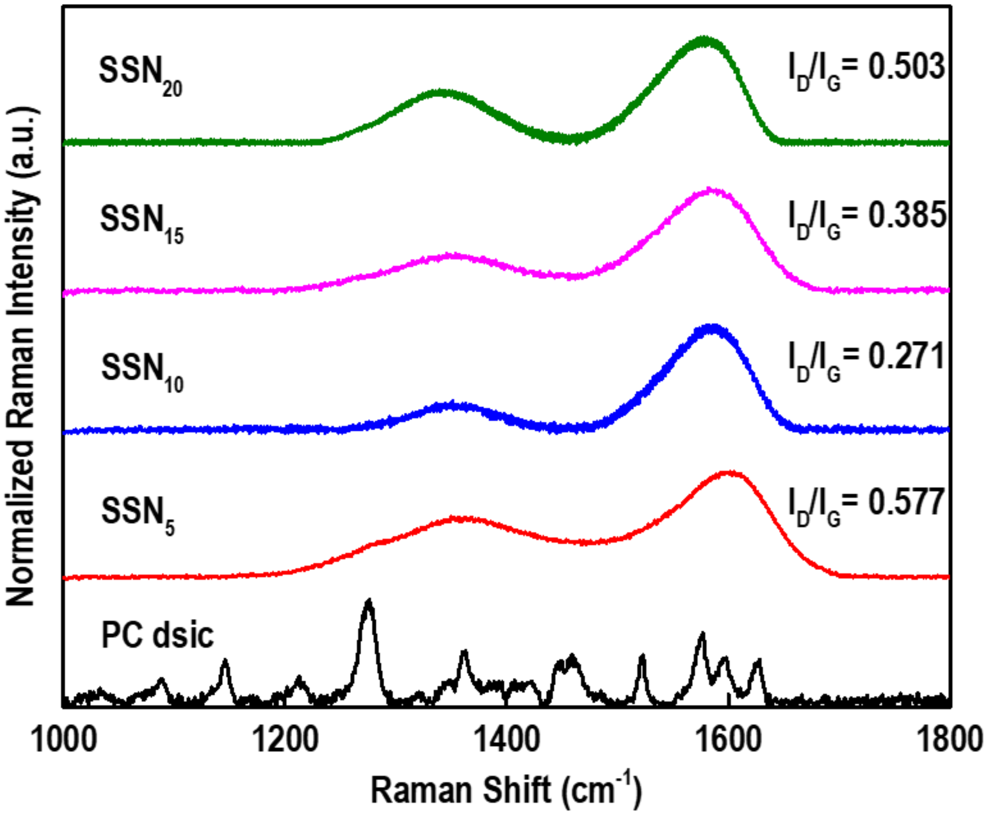

Figure S23. Raman spectra of synthesized SSN_x under different plasma treatment times.

Table S1. Surface area analysis of synthesized SSNs.

Reaction time (min)	Surface area (μm^2)
0	26.2
5	39.7
10	52.1
15	49.6
20	41.1

Table S2. Surface functional groups analysis of SSN[10].

Sample	Frequency (cm^{-1})	Assignment
PC disc (Before plasma treatment)	1566	C=C bending
	1448	C-H bending
	1413	
	1232	C-O stretching
	1195	
	1138	
	846	C=C bending
SSN	3400	-OH stretching
	2345	CO_2 stretching
	1728	C=O stretching
	1651	C=C stretching
	1585	
	1365	C-H bending
	1245	C-O stretching
	1122	
	1041	
	835	C=C bending

Table S3. Different reaction time as-synthesized SSN.

Time (min)	0	5	10	15	20
Angle (degree)	111.4	90.5	64.6	43.7	22.8

Table S4. Comparison of activity factor k and catalytic performance of the SSN and other reported catalysts in 4-NP reduction.

Catalyst	Mass of catalyst (mg)	4-NP (mmol)	Conversion time (min)	Activity factor k (s^{-1} g^{-1})	TOF (10^{-2} mmol 4-NP/mg cat. · min)	Ref.
SSN_{10}	0.0625	3×10^{-3}	0.5	3200	9.6	This work
SSN_5	0.0875	3×10^{-3}	4	56	8.57×10^{-3}	
SSN_{15}	0.05	3×10^{-3}	2	502	3	
SSN_{20}	0.04	3×10^{-3}	3	257.5	2.5	
Pd@COF-BPh	0.1	3×10^{-4}	6.08	145	5×10^{-2}	11
2.0% Ag-OH^- @DMSNs	0.15	5×10^{-4}	3	141.13	1.11×10^{-3}	12
PtRh ANMPs	0.02	4×10^{-4}	20	175	1×10^{-1}	13
$Ag_{2.6}$/C15h	0.2	2.25×10^{-4}	0.5	626.7	2.25×10^{-1}	14
rGO/LC3/AuNPs	0.03	4×10^{-3}	20	113.33	6.67×10^{-1}	15
$PdMo_2N$-TiO_2	10	6×10^{-3}	1.67	1.97	3.6×10^{-2}	16
SNTs/Pd-Fe/NC	2	6×10^{-2}	1.17	29.7	2.56	17
Ag/Fe_3O_4@GO	10	1×10^{-2}	12	0.51	8×10^{-3}	18
Ag/MR-3	2	1.25×10^{-1}	3	11.9	2.08	19
4-PtNi/C	3	2.5×10^{-4}	10	1.8	8×10^{-4}	20
Ag^0@CZ-TEB	2	5×10^{-2}	2	9.95	1.25	21
Cu_2O@ZIF-8	30	3.13×10^{-4}	14	0.157	7.44×10^{-5}	22
Cu/C	1.5	3.13×10^{-4}	2.5	17.8	8.33×10^{-3}	23
Cu/α-Fe_2O_3	1	5.63×10^{-3}	0.83	90	6.78×10^{-1}	24
Ni/C	0.05	2.95×10^{-4}	3	24780	1.97×10^{-1}	25

DE/Ni/N-C-800	3	5×10^{-4}	1.5	11.67	1.11×10^{-2}	26
$Co_{0.5}Ni_{0.5}$/C-600	0.05	3×10^{-4}	4	449.6	1.5×10^{-1}	27
MCNF@$NiCo_2O_4$	0.2	2.5×10^{-4}	45	3.02	2.78×10^{-3}	28
MoS_2/SnO_2	1.5	3×10^{-4}	13	2.8	1.54×10^{-4}	29
Pd_1/NHG	0.1	2×10^{-1}	1	926	2×10^{2}	30
AuNPs	0.003442	2×10^{-4}	2	7312.6	2.91	31
Au/g-C_3N_4	1	3.59×10^{-3}	8	7.99	4.49×10^{-2}	32
AgPd NC/rGO	0.05	3.5×10^{-4}	3	730	2.33×10^{-1}	33
Commercial Pd/C	0.05	3.5×10^{-4}	9	153.4	7.78×10^{-2}	33
AgNPs/SiNSs	20	6×10^{-3}	0.67	4.010	4.50×10^{-2}	34
AC-Ag	2	3×10^{-4}	6	3.335	2.5×10^{-3}	35
Fe_3O_4@PPyMAA/Ag	2.5	1×10^{-0}	25	0.953	1.6	36
GO-DAP-AgNPs	1	2×10^{-4}	12	0.755	1.67×10^{-3}	37
Pt@Ag	0.05	2.7×10^{-4}	8	118.334	6.75×10^{-2}	38
PtO_2/ZnO	3	2.5×10^{-4}	6.25	2.894	1.33×10^{-3}	39
RGO@AC/Pd	1	3×10^{-2}	1.65	10033.3	1.82	40
Pd_1/GDY@G-1	0.015	3.22×10^{-3}	8	230	2.68	41
Co-N_4/TiN-rGO	5	9×10^{-3}	1	19.6	1.8×10^{-1}	42
UiO-66/$Ni_{1.0}$	5	4.31×10^{-4}	4	3.167	2.16×10^{-3}	43
AgNPs/CPAN	10	1.25×10^{-1}	3	1.75	4.17×10^{-1}	44

rGO/Ni	4	1×10^{-3}	82.1	0.154	3.05×10^{-4}	45
Pt_1/NMCW	50	5.4×10^{-3}	50	0.0347	2.16×10^{-4}	46
Mn_3O_4/PdCu@NC	0.04	2.7×10^{-4}	7	132.5	9.64×10^{-2}	47
PdCo/CCF	0.15	2.7×10^{-2}	3.92	109.555	4.60	48
Pd NCs	0.14	2×10^{-4}	6	86.905	2.38×10^{-2}	49
Pd@PUN	30	2×10^{-3}	1	2.756	6.67×10^{-3}	50
MCM-41-0.15 C-H-Ni	28	7×10^{-1}	8	0.075	3.13×10^{-1}	51
PdP/FL-BP	0.008063	3×10^{-4}	0.33	10417.96	1.12×10^{1}	52
Au/graphene	1.06	2.8×10^{-4}	12	2.991	2.20×10^{-3}	53
Au@TpPa-1	20	2.7×10^{-3}	18	0.268	7.5×10^{-4}	54
H-Pd-C/N	0.045	3×10^{-4}	240	162.22	2.78×10^{-3}	55
Cu/Cu_xO@CN	0.012	2.5×10^{-4}	0.67	10500	3.12	56
Cu/BNO	5	2.5×10^{-3}	5	2.307	1×10^{-2}	57
Cu/CS-CMM	0.19685	6×10^{-4}	18	10.668	1.69×10^{-2}	58
Au@NH_2-MIL-101(Fe)	0.03	1×10^{-4}	2	1200	1.67×10^{-1}	59
Au@g-C_3N_4	2	4×10^{-4}	10	7.5	2×10^{-3}	60
Au/CeO_2@ZrO_2	0.14	3×10^{-4}	21.7	172.143	9.89×10^{-3}	61
Au@RCC3	0.2	4×10^{-4}	9	32.417	2.22×10^{-2}	62
Au/FMOF	1.5	5.76×10^{-3}	4	16.111	9.6×10^{-2}	63

Au@DMSNs	0.15	5×10^{-4}	4.5	61.111	7.41×10^{-2}	64
Au/$CaCO_3$	1	2×10^{-3}	3	0.333	6.67×10^{-2}	65

Table S5. Summary of the metal precursor price for metal-based catalyst.

Catalyst based	Metal salt	Cost per gram (US $)
Ag	$AgNO_3$	4.76
Au	$HAuCl_4$	2.79×10^2
Cu	$CuCl_2$	1.13
Pt	H_2PtCl_6	4.53×10^2
Pd	K_2PdCl_4	1.45×10^2
Fe	$FeCl_3$	9.4×10^{-1}
Co	$CoCl_2$	1.8
Ni	$NiCl_2$	7.8×10^{-1}

**The cost of the metal salt is based on the supplier's price as of September 19, 2024.*

Table S6. Comparison of reusability with other catalysts in 4-NP reduction.

Catalyst	Cycle	Conversion (%)	Reference
SSN_{10}	5	96	This work
	20	86	
PS–PDA-Ag	5	90	[66]
Fe_3O_4@PS@Ag	7	90	[67]
AgMENs	5	92	[68]
Co/PCNS	5	90	[69]
Bi@BSMX14	5	94	[70]

References

1. Luo, P.; Ji, Z.; Li, C.; Shi, G., Aryl-modified graphene quantum dots with enhanced photoluminescence and improved pH tolerance. *Nanoscale* **2013,** *5* (16), 7361-7.
2. Yang, Y.; Chen, S.; Li, H.; Yuan, Y.; Zhang, Z.; Xie, J.; Hwang, D. W.; Zhang, A.; Liu, M.; Zhou, X., Engineered Paramagnetic Graphene Quantum Dots with Enhanced Relaxivity for Tumor Imaging. *Nano Lett* **2019,** *19* (1), 441-448.
3. Flygare, M.; Svensson, K., Influence of crystallinity on the electrical conductivity of individual carbon nanotubes. *Carbon Trends* **2021,** *5*.
4. Salim, E. T.; Fakhri, M. A.; Tariq, S. M.; Azzahrani, A. S.; Ibrahim, R. K.; Alwahib, A. A.; Alhasan, S. F. H.; Ramizy, A.; Salih, E. Y.; Salim, Z. T., The unclad single-mode fiber-optic sensor simulation for localized surface plasmon resonance sensing based on silver nanoparticles embedded coating. *Plasmonics* **2024,** *19* (1), 131-143.
5. Saroj, A.; Ramanathan, V., Bismuth oxybromide based novel substrate for surface enhanced Raman spectroscopy. *Vibrational Spectroscopy* **2023,** *124*, 103463.
6. Wu, Y.; Yang, M.; Ueltschi, T. W.; Mosquera, M. A.; Chen, Z.; Schatz, G. C.; Van Duyne, R. P., SERS study of the mechanism of plasmon-driven hot electron transfer between gold nanoparticles and PCBM. *The Journal of Physical Chemistry C* **2019,** *123* (49), 29908-29915.
7. Waterhouse, G. I.; Bowmaker, G. A.; Metson, J. B., The thermal decomposition of silver (I, III) oxide: A combined XRD, FT-IR and Raman spectroscopic study. *Physical Chemistry Chemical Physics* **2001,** *3* (17), 3838-3845.
8. Stabryla, L. M.; Johnston, K. A.; Diemler, N. A.; Cooper, V. S.; Millstone, J. E.; Haig, S.-J.; Gilbertson, L. M., Role of bacterial motility in differential resistance mechanisms of silver nanoparticles and silver ions. *Nature nanotechnology* **2021,** *16* (9), 996-1003.
9. Lee, S. A.; Link, S., Chemical interface damping of surface plasmon resonances. *Accounts of chemical research* **2021,** *54* (8), 1950-1960.
10. Ţucureanu, V.; Matei, A.; Avram, A. M., FTIR spectroscopy for carbon family study. *Critical reviews in analytical chemistry* **2016,** *46* (6), 502-520.
11. Fan, M.; Wang, W. D.; Zhu, Y.; Sun, X.; Zhang, F.; Dong, Z., Palladium clusters confined in triazinyl-functionalized COFs with enhanced catalytic activity. *Applied Catalysis B: Environmental* **2019,** *257*, 117942.
12. Hu, X.-D.; Shan, B.-Q.; Tao, R.; Yang, T.-Q.; Zhang, K., Interfacial hydroxyl promotes the reduction of 4-nitrophenol by Ag-based catalysts confined in dendritic mesoporous silica nanospheres. *The Journal of Physical Chemistry C* **2021,** *125* (4), 2446-2453.
13. Yan, Q.; Wang, X.-Y.; Feng, J.-J.; Mei, L.-P.; Wang, A.-J., Simple fabrication of bimetallic platinum-rhodium alloyed nano-multipods: A highly effective and recyclable catalyst for reduction of 4-nitrophenol and rhodamine B. *Journal of colloid and interface science* **2021,** *582*, 701-710.
14. Chen, C.-S.; Chen, T.-C.; Chiu, K.-L.; Wu, H.-C.; Pao, C.-W.; Chen, C.-L.; Hsu, H.-C.; Kao, H.-M., Silver particles deposited onto magnetic carbon nanofibers as highly active catalysts for 4-nitrophenol reduction. *Applied Catalysis B: Environmental* **2022,** *315*, 121596.
15. Ren, Z.; Li, H.; Li, J.; Cai, J.; Zhong, L.; Ma, Y.; Pang, Y., Green synthesis of reduced graphene oxide/chitosan/gold nanoparticles composites and their catalytic activity for reduction of 4-nitrophenol. *International Journal of Biological Macromolecules* **2023,** *229*, 732-745.
16. Tian, X.; Zahid, M.; Li, J.; Sun, W.; Niu, X.; Zhu, Y., Pd/Mo2N-TiO2 as efficient catalysts for promoted selective hydrogenation of 4-nitrophenol: A green bio-reducing preparation method. *Journal of Catalysis* **2020,** *391*, 190-201.

17. Zhang, N.; Qiu, Y.; Sun, H.; Hao, J.; Chen, J.; Xi, J.; Liu, J.; He, B.; Bai, Z.-W., Substrate-assisted encapsulation of Pd-Fe bimetal nanoparticles on functionalized silica nanotubes for catalytic hydrogenation of nitroarenes and azo dyes. *ACS Applied Nano Materials* **2021,** *4* (6), 5854-5863.
18. Doan, V.-D.; Nguyen, N.-V.; Nguyen, T. L.-H.; Tran, V. A.; Le, V. T., High-efficient reduction of methylene blue and 4-nitrophenol by silver nanoparticles embedded in magnetic graphene oxide. *Environmental Science and Pollution Research* **2021**, 1-11.
19. Jiang, S.; Wang, L.; Duan, Y.; An, J.; Luo, Q.; Zhang, Y.; Tang, Y.; Huang, J.; Zhang, B.; Liu, J., A novel strategy to construct supported silver nanocomposite as an ultra-high efficient catalyst. *Applied Catalysis B: Environmental* **2021,** *283*, 119592.
20. Yang, X.; Wang, J.; Wei, Y.; Li, B.; Yan, W.; Yin, L.; Wu, D.; Liu, P.; Zhang, P., Cotton-derived carbon fiber-supported Ni nanoparticles as nanoislands to anchor single-atom Pt for efficient catalytic reduction of 4-nitrophenol. *Applied Catalysis A: General* **2022,** *643*, 118734.
21. Gong, W.; Wu, Q.; Jiang, G.; Li, G., Ultrafine silver nanoparticles supported on a covalent carbazole framework as high-efficiency nanocatalysts for nitrophenol reduction. *Journal of Materials Chemistry A* **2019,** *7* (22), 13449-13454.
22. Li, B.; Ma, J. G.; Cheng, P., Silica-protection-assisted encapsulation of Cu2O nanocubes into a metal–organic framework (ZIF-8) to provide a composite catalyst. *Angewandte Chemie* **2018,** *130* (23), 6950-6953.
23. Bai, Y.; Wang, Q.; Du, C.; Bu, T.; Liu, Y.; Sun, X.; Luo, W.; Li, R.; Zhao, Y.; Zheng, X., Three-dimensional Cu/C porous composite: Facile fabrication and efficient catalytic reduction of 4-nitrophenol. *Journal of colloid and interface science* **2019,** *553*, 768-777.
24. Elfiad, A.; Galli, F.; Boukhobza, L. M.; Djadoun, A.; Boffito, D. C., Low-cost synthesis of Cu/α-Fe2O3 from natural HFeO2: application in 4-nitrophenol reduction. *Journal of Environmental Chemical Engineering* **2020,** *8* (5), 104214.
25. Wu, G.; Liang, X.; Zhang, H.; Zhang, L.; Yue, F.; Wang, J.; Su, X., Highly stable and sub-3 nm Ni nanoparticles coated with carbon nanosheets as a highly active heterogeneous hydrogenation catalyst. *Catalysis Communications* **2016,** *79*, 63-67.
26. Jiang, D. B.; Liu, X.; Yuan, Y.; Feng, L.; Ji, J.; Wang, J.; Losic, D.; Yao, H.-C.; Zhang, Y. X., Biotemplated top-down assembly of hybrid Ni nanoparticles/N doping carbon on diatomite for enhanced catalytic reduction of 4-nitrophenol. *Chemical Engineering Journal* **2020,** *383*, 123156.
27. Zhao, Y.; Cao, B.; Wang, X.; Wang, X.; Al-Mamun, M.; Zhao, H.; Wang, J.; Zheng, Y.; Su, X., Facile synthesis of ultra-thin CoxNi (1-x)/C nano-sheets and their remarkable catalytic properties in 4-nitrophenol reduction. *Journal of environmental chemical engineering* **2018,** *6* (4), 5239-5248.
28. Yang, Y.; Zeng, D.; Shao, S.; Hao, S.; Zhu, G.; Liu, B., Construction of core-shell mesoporous carbon nanofiber@ nickel cobaltite nanostructures as highly efficient catalysts towards 4-nitrophenol reduction. *Journal of colloid and interface science* **2019,** *538*, 377-386.
29. Qiao, X.-Q.; Zhang, Z.-W.; Hou, D.-F.; Li, D.-S.; Liu, Y.; Lan, Y.-Q.; Zhang, J.; Feng, P.; Bu, X., Tunable MoS2/SnO2 P–N heterojunctions for an efficient trimethylamine gas sensor and 4-nitrophenol reduction catalyst. *ACS sustainable chemistry & engineering* **2018,** *6* (9), 12375-12384.
30. Hu, H.; Liu, P.; Cao, S.; You, L.; Zhang, N.; Xi, J.; Guo, S.; Zhou, K., Single Metal Atoms Anchored on N-Doped Holey Graphene as Efficient Dual-Active-Component Catalysts for Nitroarene Reduction. *Advanced Functional Materials* **2024,** *34* (12), 2307162.

31. Shen, W.; Qu, Y.; Pei, X.; Li, S.; You, S.; Wang, J.; Zhang, Z.; Zhou, J., Catalytic reduction of 4-nitrophenol using gold nanoparticles biosynthesized by cell-free extracts of Aspergillus sp. WL-Au. *Journal of hazardous materials* **2017,** *321*, 299-306.
32. Fu, Y.; Huang, T.; Jia, B.; Zhu, J.; Wang, X., Reduction of nitrophenols to aminophenols under concerted catalysis by Au/g-C3N4 contact system. *Applied Catalysis B: Environmental* **2017,** *202*, 430-437.
33. Zhu, X.-Y.; Lv, Z.-S.; Feng, J.-J.; Yuan, P.-X.; Zhang, L.; Chen, J.-R.; Wang, A.-J., Controlled fabrication of well-dispersed AgPd nanoclusters supported on reduced graphene oxide with highly enhanced catalytic properties towards 4-nitrophenol reduction. *Journal of colloid and interface science* **2018,** *516*, 355-363.
34. Yan, Z.; Fu, L.; Zuo, X.; Yang, H., Green assembly of stable and uniform silver nanoparticles on 2D silica nanosheets for catalytic reduction of 4-nitrophenol. *Applied Catalysis B: Environmental* **2018,** *226*, 23-30.
35. Shui, L.; Zhang, G.; Hu, B.; Chen, X.; Jin, M.; Zhou, G.; Li, N.; Muhler, M.; Peng, B., Photocatalytic one-step synthesis of Ag nanoparticles without reducing agent and their catalytic redox performance supported on carbon. *Journal of Energy Chemistry* **2019,** *36*, 37-46.
36. Das, R.; Sypu, V. S.; Paumo, H. K.; Bhaumik, M.; Maharaj, V.; Maity, A., Silver decorated magnetic nanocomposite (Fe3O4@ PPy-MAA/Ag) as highly active catalyst towards reduction of 4-nitrophenol and toxic organic dyes. *Applied Catalysis B: Environmental* **2019,** *244*, 546-558.
37. Nimita Jebaranjitham, J.; Mageshwari, C.; Saravanan, R.; Mu, N., Fabrication of amine functionalized graphene oxide – AgNPs nanocomposite with improved dispersibility for reduction of 4-nitrophenol. *Composites Part B: Engineering* **2019,** *171*, 302-309.
38. Lv, Z. S.; Zhu, X. Y.; Meng, H. B.; Feng, J. J.; Wang, A. J., One-pot synthesis of highly branched Pt@Ag core-shell nanoparticles as a recyclable catalyst with dramatically boosting the catalytic performance for 4-nitrophenol reduction. *J Colloid Interface Sci* **2019,** *538*, 349-356.
39. Yang, X.; Li, Y.; Zhang, P.; Zhou, R.; Peng, H.; Liu, D.; Gui, J., Photoinduced in Situ Deposition of Uniform and Well-Dispersed PtO(2) Nanoparticles on ZnO Nanorods for Efficient Catalytic Reduction of 4-Nitrophenol. *ACS Appl Mater Interfaces* **2018,** *10* (27), 23154-23162.
40. Xi, J.; Sun, H.; Wang, D.; Zhang, Z.; Duan, X.; Xiao, J.; Xiao, F.; Liu, L.; Wang, S., Confined-interface-directed synthesis of Palladium single-atom catalysts on graphene/amorphous carbon. *Applied Catalysis B: Environmental* **2018,** *225*, 291-297.
41. Li, J.; Zhong, L.; Tong, L.; Yu, Y.; Liu, Q.; Zhang, S.; Yin, C.; Qiao, L.; Li, S.; Si, R.; Zhang, J., Atomic Pd on Graphdiyne/Graphene Heterostructure as Efficient Catalyst for Aromatic Nitroreduction. *Advanced Functional Materials* **2019,** *29* (43).
42. Gu, Y.; Wu, A.; Wang, L.; Wang, D.; Yan, H.; Yu, P.; Xie, Y.; Tian, C.; Sun, F.; Fu, H., A “competitive occupancy” strategy toward Co–N4 single-atom catalysts embedded in 2D TiN/rGO sheets for highly efficient and stable aromatic nitroreduction. *Journal of Materials Chemistry A* **2020,** *8* (9), 4807-4815.
43. Hoa, L. T.; Nhi, L. T. T.; Sơn, L. V. T.; Linh, N. L. M.; Hai, H. V. M.; Khieu, D. Q.; Ersen, O., Single-Atom Ni Heterogeneous Catalysts Supported UiO-66 Structure: Synthesis and Catalytic Activities. *Journal of Nanomaterials* **2021,** *2021*, 1-16.
44. Liu, L.; Duan, Y.; Liang, Y.; Kan, A.; Wang, L.; Luo, Q.; Zhang, Y.; Zhang, B.; Li, Z.; Liu, J.; Wang, D., Cyclized Polyacrylonitrile as a Promising Support for Single Atom Metal Catalyst with Synergistic Active Site. *Small* **2022,** *18* (8), e2104142.

45. Svalova, A.; Brusko, V.; Sultanova, E.; Kirsanova, M.; Khamidullin, T.; Vakhitov, I.; Dimiev, A. M., Individual Ni atoms on reduced graphene oxide as efficient catalytic system for reduction of 4-nitrophenol. *Applied Surface Science* **2021,** *565*.
46. Tian, Z.; Deng, X.; He, P.; Wang, G.-H., Atomic Pt anchored on hierarchically porous monolithic carbon nanowires as high-performance catalyst for liquid hydrogenation. *Nano Research* **2022,** *16* (4), 5880-5886.
47. Ma, Y.; Hu, K.; Sun, Y.; Iqbal, K.; Bai, Z.; Wang, C.; Jia, X.; Ye, W., N-doped carbon coated Mn(3)O(4)/PdCu nanocomposite as a high-performance catalyst for 4-nitrophenol reduction. *Sci Total Environ* **2019,** *696*, 134013.
48. Yang, J.; Wang, W. D.; Dong, Z., PdCo nanoparticles supported on carbon fibers derived from cotton: Maximum utilization of Pd atoms for efficient reduction of nitroarenes. *J Colloid Interface Sci* **2018,** *524*, 84-92.
49. Swain, S.; M, B. B.; Kandathil, V.; Bhol, P.; Samal, A. K.; Patil, S. A., Controlled Synthesis of Palladium Nanocubes as an Efficient Nanocatalyst for Suzuki-Miyaura Cross-Coupling and Reduction of p-Nitrophenol. *Langmuir* **2020,** *36* (19), 5208-5218.
50. Yang, X.; Jiang, X.; Bashir, M. S.; Kong, X. Z., Preparation of Highly Uniform Polyurethane Microspheres by Precipitation Polymerization and Pd Immobilization on Their Surface and Their Catalytic Activity in 4-Nitrophenol Reduction and Dye Degradation. *Industrial & Engineering Chemistry Research* **2020,** *59* (7), 2998-3007.
51. Ghimire, P. P.; Zhang, L.; Kinga, U. A.; Guo, Q.; Jiang, B.; Jaroniec, M., Development of nickel-incorporated MCM-41–carbon composites and their application in nitrophenol reduction. *Journal of Materials Chemistry A* **2019,** *7* (16), 9618-9628.
52. He, Z.; Liu, R.; Xu, C.; Lai, Y.; Shan, W.; Liu, J., Black phosphorus hybridizing produces electron-deficient active sites on palladium nanoparticles for catalysis. *Applied Catalysis B: Environmental* **2021,** *285*.
53. Li, J.; Liu, C.-y.; Liu, Y., Au/graphene hydrogel: synthesis, characterization and its use for catalytic reduction of 4-nitrophenol. *Journal of Materials Chemistry* **2012,** *22* (17).
54. Pachfule, P.; Kandambeth, S.; Diaz Diaz, D.; Banerjee, R., Highly stable covalent organic framework-Au nanoparticles hybrids for enhanced activity for nitrophenol reduction. *Chem Commun (Camb)* **2014,** *50* (24), 3169-72.
55. Long, Y.; Liu, Y.; Zhao, Z.; Luo, S.; Wu, W.; Wu, L.; Wen, H.; Wang, R. Q.; Ma, J., Distinctive morphology effects of porous-spherical/yolk-shell/hollow Pd-nitrogen-doped-carbon spheres catalyst for catalytic reduction of 4-nitrophenol. *J Colloid Interface Sci* **2017,** *496*, 465-473.
56. Jia, W.; Tian, F.; Zhang, M.; Li, X.; Ye, S.; Ma, Y.; Wang, W.; Zhang, Y.; Meng, C.; Zeng, G.; Liu, J., Nitrogen-doped porous carbon-encapsulated copper composite for efficient reduction of 4-nitrophenol. *J Colloid Interface Sci* **2021,** *594*, 254-264.
57. Jiang, X.; Han, B.; Zhou, C.; Xia, K.; Gao, Q.; Wu, J., Cu Nanoparticles Supported on Oxygen-Rich Boron Nitride for the Reduction of 4-Nitrophenol. *ACS Applied Nano Materials* **2018,** *1* (12), 6692-6700.
58. Haider, S.; Kamal, T.; Khan, S. B.; Omer, M.; Haider, A.; Khan, F. U.; Asiri, A. M., Natural polymers supported copper nanoparticles for pollutants degradation. *Applied Surface Science* **2016,** *387*, 1154-1161.
59. Hu, C.; Yang, C.; Wang, X.; Wang, X.; Zhen, S.; Zhan, L.; Huang, C.; Li, Y., Rapid and facile synthesis of Au nanoparticle-decorated porous MOFs for the efficient reduction of 4-nitrophenol. *Separation and Purification Technology* **2022,** *300*.

60. Nguyen, T. B.; Huang, C. P.; Doong, R.-a., Enhanced catalytic reduction of nitrophenols by sodium borohydride over highly recyclable Au@graphitic carbon nitride nanocomposites. *Applied Catalysis B: Environmental* **2019,** *240*, 337-347.
61. Evangelista, V.; Acosta, B.; Miridonov, S.; Smolentseva, E.; Fuentes, S.; Simakov, A., Highly active Au-CeO2@ZrO2 yolk–shell nanoreactors for the reduction of 4-nitrophenol to 4-aminophenol. *Applied Catalysis B: Environmental* **2015,** *166-167*, 518-528.
62. Liu, Y.; Dong, H.; Huang, H.; Zong, W.; Miao, Y.-E.; He, G.; Parkin, I. P.; Lai, F.; Liu, T., Electron-Deficient Au Nanoparticles Confined in Organic Molecular Cages for Catalytic Reduction of 4-Nitrophenol. *ACS Applied Nano Materials* **2022,** *5* (1), 1276-1283.
63. Liu, J.; Yu, H.; Wang, L., Effective reduction of 4-nitrophenol with Au NPs loaded ultrathin two dimensional metal-organic framework nanosheets. *Applied Catalysis A: General* **2020,** *599*.
64. Ding, M.; Peng, B.; Zhou, J.-F.; Chen, H.; Zhu, Y.-S.; Yuan, E.-H.; Albela, B.; Bonneviot, L.; Wu, P.; Zhang, K., Molecular manipulation of the microenvironment of Au active sites on mesoporous silica for the enhanced catalytic reduction of 4-nitrophenol. *Catalysis Science & Technology* **2023,** *13* (7), 2001-2009.
65. Ding, Q.; Kang, Z.; Cao, L.; Lin, M.; Lin, H.; Yang, D.-P., Conversion of waste eggshell into difunctional Au/CaCO3 nanocomposite for 4-Nitrophenol electrochemical detection and catalytic reduction. *Applied Surface Science* **2020,** *510*.
66. Song, Y.; Jian, M.; Qiao, L.; Zhao, Z.; Yang, Y.; Jiao, T.; Zhang, Q., Efficient removal and recovery of Ag from wastewater using charged polystyrene-polydopamine nanocoatings and their sustainable catalytic application in 4-nitrophenol reduction. *ACS Applied Materials & Interfaces* **2024,** *16* (5), 5834-5846.
67. Wang, Y.; Gao, P.; Wei, Y.; Jin, Y.; Sun, S.; Wang, Z.; Jiang, Y., Silver nanoparticles decorated magnetic polymer composites (Fe3O4@ PS@ Ag) as highly efficient reusable catalyst for the degradation of 4-nitrophenol and organic dyes. *Journal of Environmental Management* **2021,** *278*, 111473.
68. Yeh, Y.-J.; Chiang, W.-H., Ag microplasma-engineered nanoassemblies on cellulose papers for surface-enhanced Raman scattering and catalytic nitrophenol reduction. *ACS Applied Nano Materials* **2021,** *4* (6), 6364-6375.
69. Hu, L.; Liu, X.; Guo, A.; Wu, J.; Wang, Y.; Long, Y.; Fan, G., Cobalt with porous carbon architecture: Towards of 4-nitrophenol degradation and reduction. *Separation and Purification Technology* **2022,** *288*, 120595.
70. Ghosh, K.; Roy, S. S.; Giri, P., Fast Reduction of 4-Nitrophenol and Photoelectrochemical Hydrogen Production by Self-Reduced Bi/Ti3C2T x/Bi2S3 Nanocomposite: A Combined Experimental and Theoretical Study. *ACS Applied Materials & Interfaces* **2024,** *16* (32), 42007-42020.
